# Luck perception is associated with less frequent preventive practices and a higher number of social contacts among adults during the SARS-CoV-2 pandemic

**DOI:** 10.1016/j.puhip.2022.100325

**Published:** 2022-10-08

**Authors:** Arata Hidano, Bethan Page, James W. Rudge, Gareth Enticott

**Affiliations:** a Communicable Disease Policy Research Group, Faculty of Public Health and Policy, London School of Hygiene and Tropical Medicine, United Kingdom; b Faculty of Public Health, Mahidol University, Bangkok, Thailand; c School of Geography and Planning, Cardiff University, United Kingdom

**Keywords:** Locus of control, Luck, Fatalism, Covid-19, Contact survey, Health behaviour, HLC, Health Locus of Control, NB, Negative binomial, aOR, Adjusted odds ratio, CR, Count ratio

## Abstract

**Objectives:**

Non-pharmaceutical interventions have been crucial to reduce transmission of the SARS-CoV-2 virus in many countries including the United Kingdom. A key research priority has been to better understand psychological and social determinants of health behaviours. We aimed to quantify the impact of luck perception on contact and preventive behaviours among adults in the UK, adjusting for key confounders.

**Study design:**

A cross-sectional study.

**Methods:**

Data were collected between July 28 and August 31, 2020. Luck perception, which refers to a belief whether individual's SARS-CoV-2 infection status is determined by fate or chance, was measured using Chance score, drawing on Health Locus of Control Theory. Self-reporting online questionnaires were administered to obtain participants' contact patterns and frequencies of avoiding crowds, hand washing and wearing a mask. Associations between luck perception and protective behaviours and contact patterns were quantified using regression models.

**Results:**

Data from 233 survey respondents were analysed. Chance score was negatively associated with all protective behaviours; avoiding crowds (adjusted odds ratio (aOR) 0.46, 95% confidence interval (CI) 0.25–0.86, p = 0.02), washing hands (aOR 0.35, 95%CI 0.17–0.70, p = 0.003), and wearing masks (aOR 0.58, 95%CI 0.34–0.99, p = 0.046). For non-physical contacts (with or without distancing), a significant interaction was identified between Chance score and ethnicity. Chance score increased the number of non-physical contacts among white British, an opposite trend was observed for non-white participants.

**Conclusions:**

Luck perception during the pandemic may affect individuals’ health protection behaviours and contact patterns. Further mechanistic understandings of human behaviours against infectious diseases are indispensable for effective response to future pandemics.

## 1 Introduction

Mathematical modelling has played a pivotal role in guiding public health policies in many countries during the SARS-CoV-2 pandemic. These models typically use human contact patterns as one of the key inputs. Data from information technology companies such as Google have highlighted that human mobility and social mixing patterns can change drastically over the course of the pandemic. Nevertheless, we still lack mechanistic models that explain these changes in response to epidemic status, policies and other socio-psychological factors [1]. As a result, an accurate forecasting of the trends of SARS-CoV-2 cases ahead of time remains challenging.

During unprecedented and highly uncertain circumstances, sense of helplessness and lack of control have been observed as potentially important determinants of compliance with public health guidance [2]. Luck perception refers to belief that one's health is controlled by luck, chance, fate or God [3], and one of three dimensions of the Health Locus of Control (HLC) theory. Previous studies reported higher luck perception is associated with various health behaviours such as eating less healthy diet and smoking [4]. Less studied is that the impact of luck perception on health behaviours for acute infectious diseases [2] and, to our knowledge, no studies have evaluated its impact on human contact patterns. We hypothesised that individuals with greater luck perception were less engaged in protective behaviours for SARS-CoV-2. Our objective was therefore to quantify the impact of luck perception on human contact patterns and other preventive behaviours during a period of the SARS-CoV-2 pandemic.

## 2 Methodology

Data were collected using an online survey between July 28 and August 31, 2020. A non-random sample was recruited using online methods. Participants had to be aged 18 and over currently living in the UK. The survey comprised of 25 questions, pertaining to the participant's demographics (age, gender, ethnicity and marital status) and socio-economic status, work and employment status, HLC and trust towards the government, health behaviours and contact patterns, and SARS-CoV-2 related information (see Supplementary material). HLC is measured across three domains; Internal, Powerful others and Chance [3]. Individuals who have Chance locus of control would believe their health is largely determined by chance. HLC was measured using three items for each domain (and their averages were calculated) and participants could answer each item using a five-point scale ranging from 1: ‘strongly disagree’ to 5: ‘strongly agree’. We collected the contact information using the POLYMOD survey design, which has been widely used to collect human contact data [5]. In short, participants were asked to report the number of contacts they made (except with individuals they lived with) for three types of direct contacts on the day prior to the survey: physical contact is any sort of skin-to-skin contact, while non-physical contact is a contact that involves exchanging a few words face-to-face with or without 1 m distancing. We also asked the frequency of three health behaviours using a five-point scale; avoiding crowds, washing hands, and mask wearing. We categorised answers ‘Always’ for health behaviour questions as outcome positive and otherwise negative.

Chance score variable was the exposure of interest. Internal and Powerful others variables were treated as potential confounders. For each of the six outcome variables, a separate regression model was constructed. Univariable analyses were conducted by fitting a logistic regression and Poisson regression model for the health behaviour outcomes and the number of contacts for each type, respectively. Log-likelihood ratio test (LRT) was conducted for each explanatory variable and p-value computed. We first added Chance variable and two a priori confounder variables, age and gender, in each model. At this stage LRT was conducted to compare the fit of Poisson and negative-binomial (NB) regression for models in which the outcome variable was number of contacts. LRT showed NB model fitted better for all three contact outcomes, hence NB model was used hereafter. Multivariable analyses began by adding other all confounder variables (i.e. no screening was conducted), one at a time, in the order of their univariable p-values using the forward selection procedure. All SARS-CoV-2 related variables except current symptom, the result of diagnostic tests, and whether those living in the same household received tests, were not included in this analysis because they were considered intermediate variables between Chance and the outcomes. Potential confounders were retained if LRT showed p < 0.05 for the added variable. This process was repeated until no more variables were added. Finally, two-way interactions were examined for between Chance variable and other variables and interaction terms with p ≤ 0.1 in LRT were included in the model unless the inclusion caused complete or quasi-complete separation. The results of NB models were described as count ratio, indicating how many times the number of contacts increases by a unit increase in Chance score. Estimated effects of Chance variable that included interaction terms were shown, where applicable, visually by predicting the probability and count of the outcome for the logistic and NB model, respectively.

Three sensitivity analyses were carried out for the preventive practices. Firstly, the impact of using a different cut-off point for the health behaviour outcome was examined; the binary outcome was re-categorised and defined positive if individuals responded ‘Often’ or ‘Always’. This was, however, infeasible for washing hands as these two categories dominated (92.2%) the data. The final multivariable models were fitted for the new outcome variables and coefficients re-estimated. In addition, a proportional odds model was fitted for each of the health behaviours in which the outcome had three categories; ‘Always’ was coded as ‘3’, ‘Often’ as ‘2’ and other categories (i.e. ‘Sometimes’, ‘Occasionally’ and ‘Never’) as 1. Explanatory and confounder variables, and interactions terms (if any) included were the same as those in the original logistic models. Thirdly, the final multivariable logistic models were run for the data obtained before August 8, 2020 and coefficients computed because the requirement of wearing masks was extended to more indoor settings from August 8.

All statistical analyses were carried out using R version 4.1.1.

## 3 Result

A total of 233 eligible responses were obtained. Mean numbers of physical contacts, non-physical contacts without distancing and with distancing were 0.57 (95% confidence interval (CI): 0.38–0.76), 1.86 (95%CI: 1.17–2.56) and 3.64 (95%CI: 2.76–4.52) times per day, respectively. The mean number of contacts for individuals between 18 and 59 years old in the UK population adjusted by age and gender was estimated to be 6.23 (95%CI 5.66–6.63). The adjusted mean numbers of contacts for individuals working full-time and part-time worker were 5.89 (95%CI 4.77–7.25) and 9.93 (95%CI 8.20–11.77), respectively.

The number of physical contacts was not associated with Chance score in the multivariable analysis (Count ratio (CR) 0.90, 95%CI 0.58–1.39, p = 0.62). On the other hand, for both non-physical contacts with and without distancing, higher Chance score was associated with a larger number of contacts. The final multivariable models for these two contact types included an interaction term between Chance score and ethnicity (p = 0.05 and p = 0.006, respectively). For white British, a unit increase in Chance score increased the number of non-physical contacts without distancing and distancing 1.42 (95%CI 0.94–2.13, p = 0.1) and 1.43 (95%CI 1.03–1.98, p = 0.03) times, respectively. For non-white groups, an opposite association was identified, where a unit increase in Chance score substantially reduced the number of these contacts (CR 0.15 95%CI 0.03–0.85 for without distancing and CR 0.13 95%CI 0.03–0.57 for distancing). No associations were identified for white non-British.

Chance score was negatively associated with all the health behaviour variables both in the univariable and the multivariable analysis (Table 1); the adjusted odds ratio (aOR) was 0.46 (95%CI 0.25–0.86) for avoiding crowds, 0.35 (95%CI 0.17–0.70) for washing hands, and 0.58 (95%CI 0.34–0.99) for wearing masks. No interactions were identified between Chance score and other confounding variables. Sensitivity analyses showed that the change in the cut-off and the exclusion of responses after August 8 resulted in a quantitative change in the coefficient of Chance score but generally the negative associations remained.

**Table 1:** Odds ratios (ORs) and their 95% confidence interval of Chance score for protective behaviours.

Outcome behaviours		OR	2.5%	97.5%	P value
Avoiding crowds	Crude^a^	0.61	0.37	1.02	0.06
	Adjusted^b^	0.46	0.25	0.86	0.02
	Sensitivity 1^c^	0.76	0.47	1.22	0.26
	Sensitivity 2^d^	0.57	0.29	1.11	0.09
	Sensitivity 3^e^	0.64	0.42	0.98	0.04
Wash hands	Crude^a^	0.66	0.40	1.06	0.09
	Adjusted^f^	0.35	0.17	0.70	0.003
	Sensitivity 1^c^	NA			
	Sensitivity 2^d^	0.36	0.16	0.80	0.01
	Sensitivity 3^e^	0.34	0.18	0.66	0.001
Wear masks	Crude^a^	0.55	0.34	0.90	0.02
	Adjusted^g^	0.58	0.34	0.99	0.04
	Sensitivity 1^c^	0.86	0.55	1.36	0.52
	Sensitivity 2^d^	0.44	0.23	0.82	0.01
	Sensitivity 3^e^	0.72	0.49	1.07	0.1

a Crude estimate from univariable analysis.

b Adjusted estimate controlling for Age, Gender, Work status, Live with people aged 5y-18y

c Sensitivity analysis by changing the cut-off for outcomes (except for washing hands).

d Sensitivity analysis by using data collected before August 8, 2020.

e Sensitivity analysis using multinominal proportional odds model.

f Adjusted estimate controlling for Age, Gender, Work status, Key worker status, Employment status, Live with people aged 4y or younger, Live with people aged 5y-18y, Currently have shortness of breath.

g Adjusted estimate controlling for Age, Gender, Key worker status, Income status, Principal component 1 for trust, Powerful others score.

## 4 Discussion

While many studies examined the association between health behaviours and social and individual factors such as trust and risk perception [6], no studies have quantified the impact of luck perception on human contact patterns. Drawing on Health Locus of Control Theory, and using sets of well-validated methods, we provide preliminary evidence that higher luck perception may be associated with a greater contact frequencies as well as less frequent protective behaviours, at least based on self-reported frequencies, during the SARS-CoV-2 pandemic.

We employed POLYMOD approach which has been used widely to generate contact pattern data essential for mathematical modelling studies and provide more objective quantitative behavioural data compared to other type of questions such as participants’ intention to perform behaviours [5]. With limited health practices and more frequent contacts, individuals with greater luck perceptions may play an important role in spreading the disease. This association seems to vary across ethnic groups, however, and luck perception was associated with fewer non-physical contacts among non-white participants. Previous studies suggested that luck perception and its closely linked concept, fatalism, have different meanings and effects on health behaviours across cultures, religions, contexts and structures [7]. While this might explain the differential effect of luck perception across ethnic groups observed in our study, great caution is needed in interpreting these results given small sample sizes for each ethnic group and potential selection and/or reporting biases as discussed below.

We reason that collider bias has not substantially distorted our results. Collider bias would occur in this study if participation was associated with likelihood of implementing health behaviours and/or number of contacts (Condition 1) and with luck perception (Condition 2). Condition 1 could occur because individuals with greater consciousness in health may have participated in this study with a greater probability. However, our study population was comparable in terms of health behaviours to that in the CoMix survey, which recruited representative samples of the UK population by gender and age. When adjusting for the demographic structure of the UK population, we estimated the mean number of contacts among age 18–59 years old to be 5.89 for full-time and 9.93 for part-time workers, which are within the 95%CI of the CoMix estimate for mid-August 2020 [8]. Furthermore, 58% of our participants reported they wore a mask often or always, which is consistent to the CoMix study that reported a similar number (around 60%).

Overall, our result is consistent to previous findings that individuals with higher luck perception are less likely to be engaged in health behaviours that are associated with chronic diseases [4]. One study reported that higher luck perception slightly decreased the adherence to SARS-CoV-2 sanitary protocols [9]. A recent study showed that higher perceived efficacy of a specific protective behaviour against SARS-CoV-2 is associated with a greater likelihood of implementing this behaviour. Given the perception of lack of control is widely associated with perceived efficacy and behavioural efficacy [10], more in-depth studies should inquire how various factors formulate these perceptions such as poverty and access to healthcare systems in general and during the pandemic.

## 5 Conclusions

We showed that luck perception may be a determinant of implementation of health protective behaviours and social contact patterns during infectious disease epidemics. Despite widespread availability and use of data on human mobility and contact patterns during the SARS-CoV-2 pandemic, our mechanistic understanding of individual and social factors influencing human contact and behavioural change remains limited. Studies on these factors during peacetime are indispensable for effective response to future pandemics.

## Ethical approval

Ethical approval was obtained from both LSHTM (Reference 21877) and Cardiff University to conduct the survey.

## Funding

This research did not receive any specific grant from funding agencies in the public, commercial, or not-for-profit sectors.

## Competing interests

None to declare.

## References

[1] Bedson J., Skrip L.A., Pedi D., Abramowitz S., Carter S., Jalloh M.F. (2021 Jun 28). A review and agenda for integrated disease models including social and behavioural factors. Nat. Human Behav..

[2] Akesson J., Ashworth-Hayes S., Hahn R., Metcalfe R.D., Rasooly I. (2020 May). https://www.nber.org/papers/w27245.

[3] Wallston K.A., Strudler Wallston B., DeVellis R. (1978 Mar 1). Development of the multidimensional health locus of control (MHLC) scales. Health Educ. Monogr..

[4] Cheng C., Cheung M.W.L., Lo B.C.Y. (2016 Oct 1). Relationship of health locus of control with specific health behaviours and global health appraisal: a meta-analysis and effects of moderators. Health Psychol. Rev..

[5] Mossong J., Hens N., Jit M., Beutels P., Auranen K., Mikolajczyk R. (2008 Mar 25). Social contacts and mixing patterns relevant to the spread of infectious diseases. PLoS Med..

[6] Kojan L., Burbach L., Ziefle M., Calero Valdez A. (2022 Mar 24). Perceptions of behaviour efficacy, not perceptions of threat, are drivers of COVID-19 protective behaviour in Germany. Humanit. Soc. Sci. Commun..

[7] Perfetti A.R. (2018 Mar 1). Fate and the clinic: a multidisciplinary consideration of fatalism in health behaviour. Med. Humanit..

[8] Gimma A., Munday J.D., Wong K.L., Coletti P., Zandvoort K van, Prem K. (2021 May). https://www.medrxiv.org/content/10.1101/2021.05.28.21257973v1.

[9] Nordfjaern T., Mehdizadeh M., Fallah Zavareh M. (2021). Social psychology of coronavirus disease 2019: do fatalism and comparative optimism affect attitudes and adherence to sanitary protocols?. Front. Psychol..

[10] Gellman M.D. (2020). Encyclopedia of Behavioral Medicine.

