# Supplementary material for: Luck perception is associated with less frequent preventive practices and a higher number of social contacts among adults during the SARS-CoV-2 pandemic
Source: Public Health Pract (Oxf). 2022 Oct 8;4:100325. doi: 10.1016/j.puhip.2022.100325 (PMC9546777; doi:10.1016/j.puhip.2022.100325)
Supplement: Multimedia component 1 [file mmc1.docx]

Supplementary material

**1. Theory**

*1.1 Health locus of control*

The Health Locus of Control (HLC) is a psychological and behavioural theory which describes the extent to which an individual believes that their health is within their control. The Multidimensional HLC (MHLC) scale was developed in the 1970s from Rotter’s Social Learning Theory about individuals perceiving outcomes as within their control more generally ^1,2^. Individuals that have an internal locus of control believe that the locus, or ‘place’, where control over their health is situated, is within themselves. Individuals whose locus of control is external to themselves believe that their health is largely determined by chance, or by powerful others, such as doctors. HLC is therefore measured across three domains; Internal, Powerful others and Chance ^2,3^. Importantly, these three domains are considered independent in MHLC and it may be possible that individuals have high Internal and Chance scores ^4^.

Previous studies have reported HLC is influenced by socio-demographic factors such as gender, age and education ^3,5^ and own experiences and reinforcement history such as illness ^6^. While this suggests HLC is an individual cognitive construct whereby an intervention would involve attempts to change one’s beliefs ^7^, HLC is shaped by wider social and contextual factors such as neighbourhood socio-economic status and social structure ^3,5^.

*1.2 Dimension of trust towards government*

Trust has been defined as “accepted vulnerability to another’s possible but not expected ill will (or lack of good will)” ^8^. In health contexts, this vulnerability is due to information asymmetry creating a power imbalance between the trusting and the trusted parties ^9^. There is debate within social science literature about whether trust is a multidimensional concept ^10,11^, and if it is, what those dimensions are. For example, in a study which aimed to explore the different dimensions of trust using principal component analysis ^11^, it was found that trust could best be explained by two components, a ‘generalised trust’ component and a ‘scepticism’ component. The ‘generalised trust’ component encompassed the concepts of ‘competence’, ‘care’, ‘fairness’ and ‘openness’. ‘Competence’ portends to the perceived expertise of the individual or institution. ‘Fairness’ relates to how different individuals or sections of society’s viewpoints are taken into consideration, and ‘openness’ relates to honesty and transparency. ‘Care’ relates to the perception that the trusted party acts in the best interests of the trusting party. The ‘scepticism’ component encompassed the constructs of ‘reliability’, that is how consistent a trusted party is, ‘integrity’, that is whether they are influenced by ‘vested interests’, and also the ‘credibility’ of the information provided by trusted parties. As articulated by Chan ^12^, while ‘generalised trust’ refers to a cognitive judgement of trustee (e.g. government), ‘scepticism’ represents an affective dimension of trust.

**2. Method**

*2.1.1 Measure of health locus of control and trust towards government*

Individuals’ health locus of control (HLC) were measured using three items each for Chance, Internal and Powerful others dimensions. Participants could answer each item using a five-point scale ranging from 1: ‘strongly disagree’ to 5: ‘strongly agree’. Scores for three items in each dimension were averaged and used as a continuous variable. Trust towards and confidence in the government was measured using 15 items. These items covered seven dimensions of trust previously proposed ^10,13^ and one item related to value similarity ^11^. These seven dimensions included openness, reliability, integrity, credibility, fairness, caring, and competence. Value similarity has been proposed as an important determinant of individuals’ trust judgement under complex circumstances ^14^, however, a previous study reported this variable explained only little of the variation of trust once the seven dimensions were considered ^11^. These items also had a five-point scale and analysed using Principal component analysis (PCA) as described below.

*2.1.2 Health behaviours and contact*

Participants were asked about the frequency of four health behaviours in the past seven days using a five-point scale ranging from 1: ‘Never’ to 5: ‘Always’. The following statements were used: “I avoid going to crowded places (e.g. supermarkets, high street stores)”, “If I go out/leave my home, I wash my hands when I get home”, “I wear a mask when I go outside”, and “I try not to meet people when I feel sick”.

Participants were also asked to report the number of contacts they made for three types of direct contacts on the day prior to the survey. We defined each type of direct contact as follows. Physical contact is any sort of skin-to-skin contact, while non-physical contact is a contact that involves exchanging a few words face-to-face with or without 1m distancing. We asked participants to consider contacts with individuals that they did not live with. We did not collect information about contacts with household members as this was considered inappropriate by the pilot participants. These data were collected by adopting and slightly modifying the POLYMOD contact survey design, which has been widely used to collect human contact data before ^15–17^ and during the SARS-CoV-2 pandemic ^18,19^. Participants were first asked to report the number of each type of contact with those they were not living with at the time of the survey (‘group contacts’), then to list each contact and characteristics including age and sex of individuals they met, location and duration of the meeting (‘individual contacts’). If they met more than five individuals, we asked them to list five individuals they randomly chose.

*2.1.3 SARS-CoV-2 related information*

Participants were asked if they thought they had been infected with SARS-CoV-2 and why they thought so. Participants were then asked to report whether they have received any diagnostic tests for SARS-CoV-2, and where applicable the test date, type and result. The number of housemates who received diagnostic tests and their results were also captured. Participants were then asked to choose any symptoms they have had in the past seven days. We then asked participant’s trust in the SARS-CoV-2 diagnostic test using a five-point scale ranging from ‘Don’t trust the result at all’ to ‘Strongly trust the result’. Finally, participants were asked to report how likely they thought they were to become infected with SARS-CoV-2 in the next four weeks on a scale of 1 (‘Not likely at all’) to 10 (‘Extremely likely’).

**3. Statistical analysis**

All statistical analyses were carried out using R version 4.1.1.

*3.1 Descriptive statistics*

We categorised answers ‘Always’ for health behaviour questions as outcome positive and otherwise negative. (The cut-off for binarization of these variables was also explored in a sensitivity analysis, described in section 3.2.4). The health behaviour “I try not to meet people when I feel sick” was dropped from the subsequent analysis as more than 90% of participants answered ‘Always’ to this question. We calculated the proportions of the outcome positive stratifying by each demographic, socio-economic, and work and employment status. The mean number of contacts, summing over three contact types, and associated confidence intervals were computed. Additionally, to make these statistics comparable to those from the large scale contact survey implemented in a similar study period ^18^, we estimated the mean number of contacts for adults (18 to 59 years old) in the 2020 UK population using the approach taken by this previous study. Briefly, we calculated the sampling weight of each study participant by dividing the proportion of a given age group and gender in the UK population ^20^ by the number of observations with the same age and gender group in our dataset. Individuals who identified as other than male or female in this study were removed from this calculation. We used age band 55 – 64 years old rather than two distinct bands (55 -59 and 60 – 64 years old) in our survey, therefore we assumed those within this age group in our study were under 60 years old for this calculation. We then sampled 1000 individuals with replacement from our dataset using these weights to compute the mean number of contacts. A bootstrap confidence interval was computed by repeating this process 1000 times. These statistics were also computed for individuals working full-time (including students) and part-time.

A previous study on UK contact patterns censored the maximum number of contacts at 50, so that individuals with very high numbers of contacts did not substantially affect the analysis ^18^. We adopted this approach and censored two observations that reported the number of non-physical contacts with distancing greater than 50.

*3.2 Principal component analysis (PCA)*

All 15 variables related to trust and confidence towards the government’s approach towards SARS-Cov-2 were entered into PCA analysis. PCA was first conducted using prcomp function, which uses the spectral decomposition approach, to identify the number of components which have eigenvalue larger than 1. All 15 variables were centred and scaled. Subsequently, varimax rotation was conducted using function principal, to improve the interpretability of PCA analysis. We also computed a mean of all 15 trust scores after reversing the scores of four questions in which higher scores represented greater distrust.

*3.3 Sensitivity analysis*

Two sensitivity analyses were carried out for the preventive practices. Firstly, the impact of using a different cut-off point for the health behaviour outcome was examined; the binary outcome was re-categorised and defined positive if individuals responded ‘Often’ or ‘Always’. This was, however, infeasible for washing hands as these two categories dominated (92.2%) the data. The final multivariable models were fitted for the new outcome variables and coefficients re-estimated. In addition, a proportional odds model was fitted for each of the health behaviours in which the outcome had three categories; ‘Always’ was coded as ‘3’, ‘Often’ as ‘2’ and other categories (i.e. ‘Sometimes’, ‘Occasionally’ and ‘Never’) as 1. Explanatory and confounder variables, and interactions terms (if any) included were the same as those in the original logistic models. Secondly, the final multivariable logistic models were run for the data obtained before 8^th^ August 2020 and coefficients computed because the requirement of wearing masks was extended to more indoor settings from 8^th^ August ^21^.

*3.4 Prediction of Chance scores*

A linear regression model was developed using Chance variable as an outcome. Multivariable models were constructed following the same modelling process explained as above, forcing age and gender into the model as *a priori* confounder, except variables with p ≤ 0.1 in LRT was included in the final model. Multiple corresponding analysis was conducted for the variables included in the final model to visualise associations between these variables. Chance score was predicted for each covariate pattern.

**4. Results
4.1 Data**

A total of 234 responses were obtained. One observation was removed because it was a response from outside the UK. The majority (61%) of responses were obtained in the first week of the survey launching. Individuals between 25 – 34 years old accounted for 38.1% of respondents, while this age group comprises 12% of the UK population. Median values for Chance, Internal and Powerful others health locus of control (HLC) variables were 2, 3.3, and 3.7, respectively (Supplementary Figure S1). While there was no statistically significant correlation between Chance and Internal variables (Spearman correlation coefficient (r) = 0.07, p = 0.27) and Chance and Powerful others variables (r = -0.08, p = 0.26), Internal and Powerful others variables had a moderate correlation (r = 0.19, p = 0.003). There was a significant negative correlation between the score for the perceived likelihood of becoming infected with SARS-CoV-2 and Internal score (r = -0.22, p < 0.001), while no statistical associations were found for other two HLC variables (Supplementary Figure S2). The median trust score (which was based on the mean of 15 trust variables for each participant) was 2.4, with range 1 – 4.8, across participants (Supplementary Figure S1).

The majority (78.8%) of participants reported “Always” to washing hands over the past seven days, compared with 26.0% and 20.3% for wearing masks and avoiding crowds, respectively (Figure S3 A-C). Based on ‘group contacts’ data, mean number of physical contacts, non-physical contacts without distancing and with distancing were 0.57, 1.86 and 3.64, respectively (Figure S3 D-F). A total of 421 contacts were reported by 190 individuals based on ‘individual contacts’ data. Contacts at workplaces accounted for 4.1%, 21.6% and 14.4% of physical, non-physical without distancing and non-physical with distancing, respectively. Majority of contacts occurred at settings other than workplace, school and medical settings (Figure S4 A). When stratified by the median Chance score, those in the upper 50^th^ percentile Chance score (Figure S4 C) had a higher proportion of non-physical contacts without distancing at other indoor settings (67.7%) than those in the lower 50^th^ percentile (46%, Figure S4 B).

Seventy-two percent of the participants were female and proportions of individuals avoiding crowds and washing hands were higher for female than male. In terms of ethnicity, white British was dominant (80.1%), followed by non-British white and other ethnicity. More than half (52.8%) of the participants had a postgraduate level education. A trend was observed that key-workers involved in health or social care implemented less frequently the health behaviours and had a larger number of contacts compared to non-key workers (Table S1 and S2). Participants working part-time had a greater number of contacts (unadjusted mean 8.0) compared to full-time working participants (unadjusted mean 4.7). The mean contacts largely varied across different working status groups, where those working from home had a low number of contact (unadjusted mean 2.4) and those working as usual as before the pandemic had a larger number of contact (unadjusted mean 11.4). The mean number of contacts for individuals between 18 – 59 years old in the UK population adjusted by age and gender was estimated to be 6.23 (95%CI 5.66 – 6.63). The adjusted mean numbers of contacts for individuals working full-time and part-time worker were 5.89 (95%CI 4.77 – 7.25) and 9.93 (95%CI 8.20 – 11.77), respectively.

**Figure S1. Distributions of Health locus of control (HLC) scores**


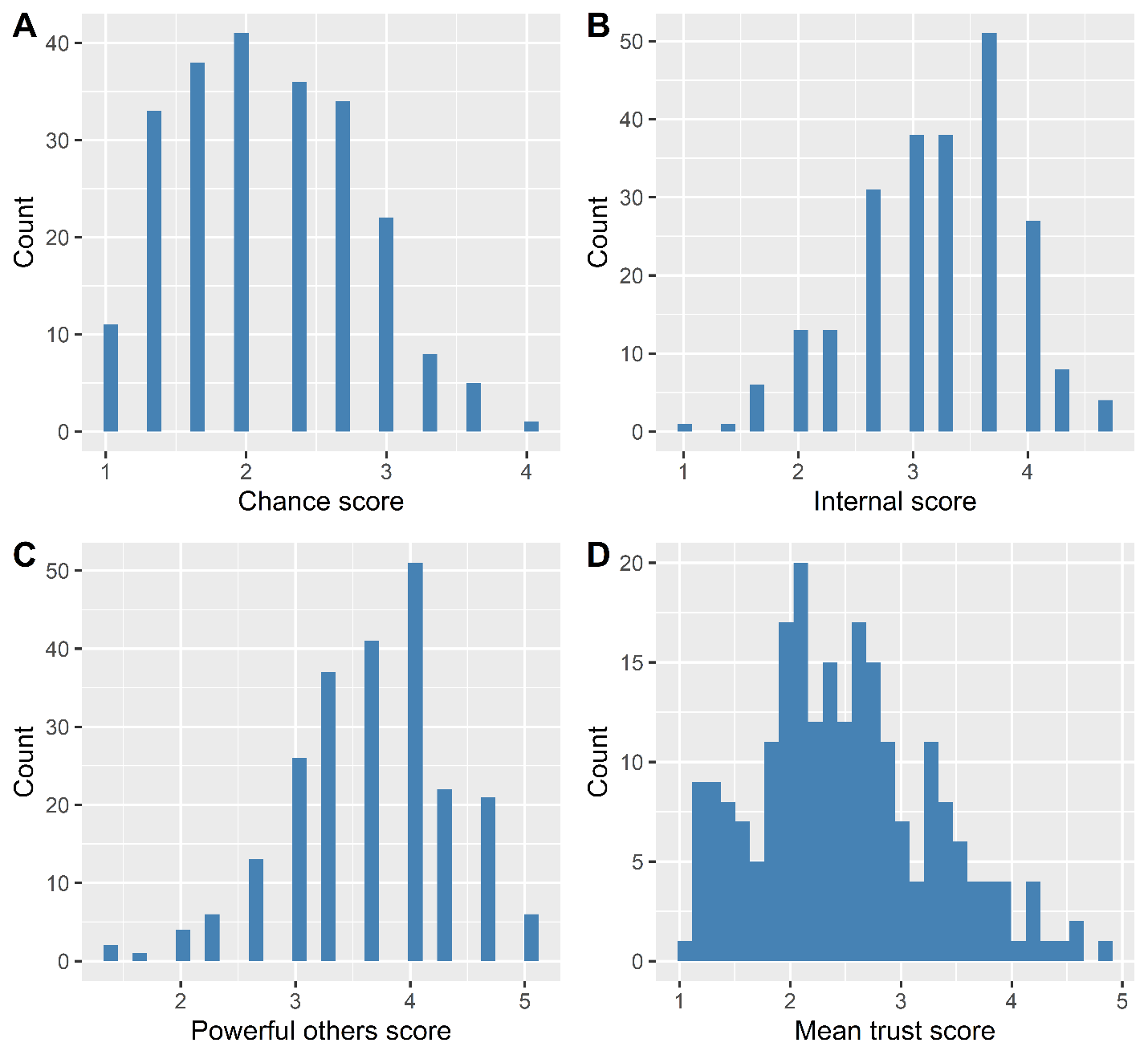


**Figure S2. Associations between the perceived likelihood of becoming infected with SARS-CoV-2 in the next four weeks and HLC scores**


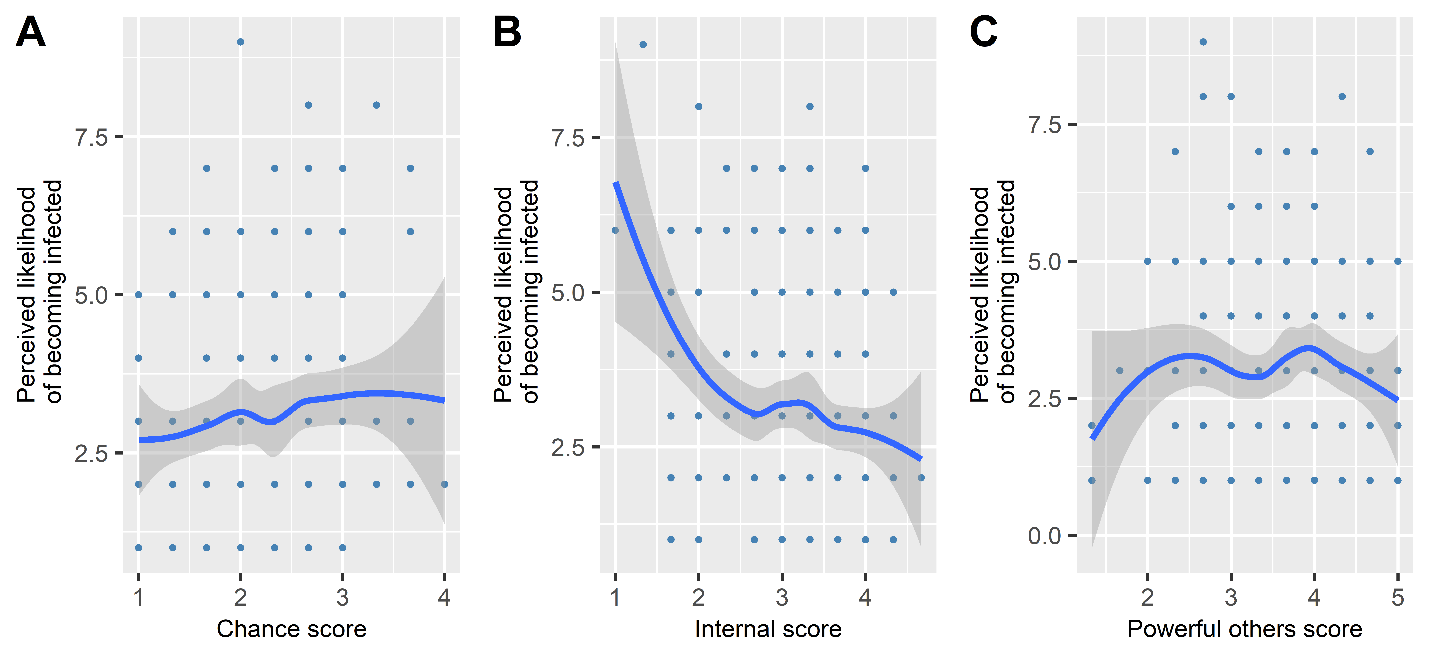


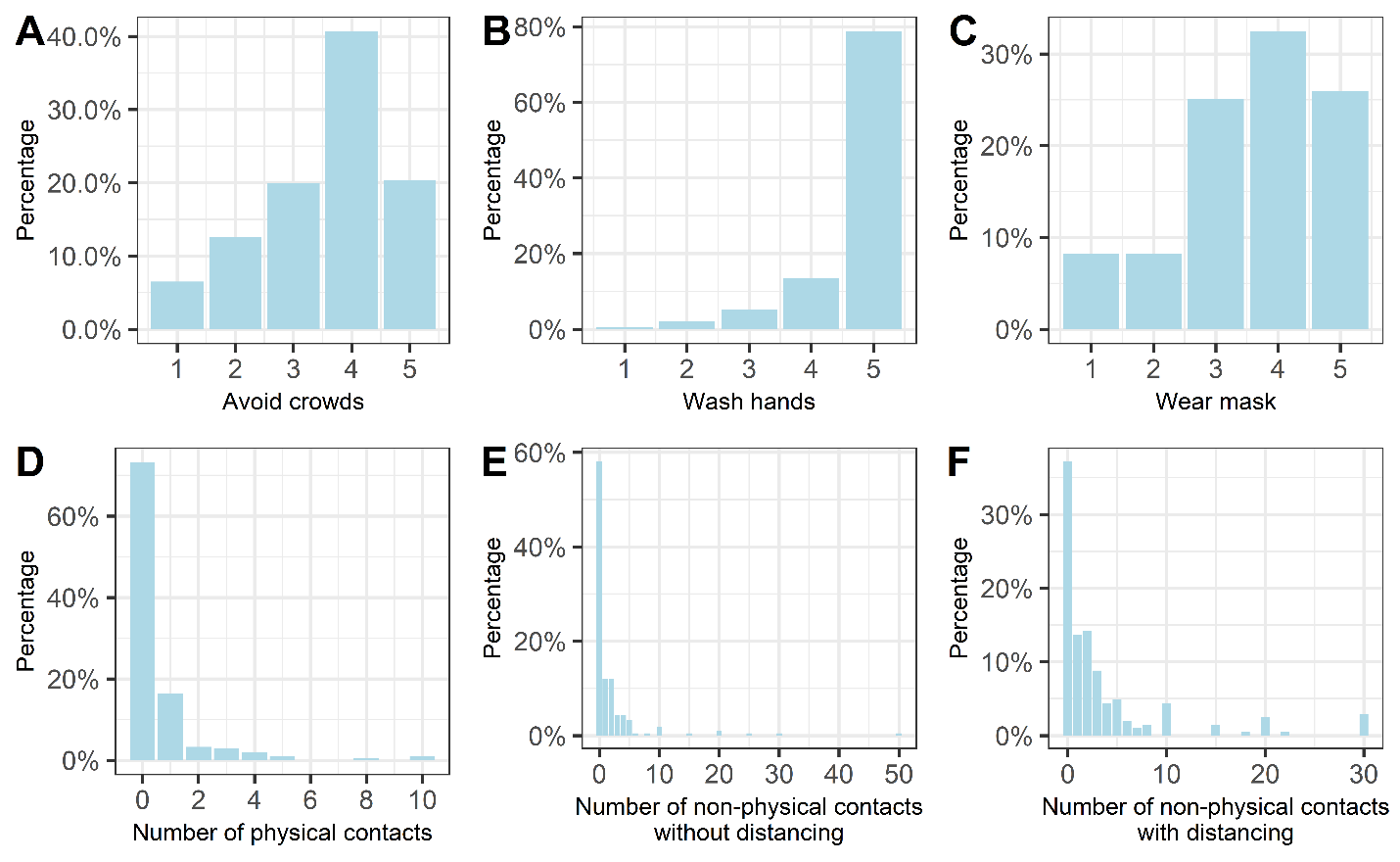
**Figure S3. Distributions of behavioural and contact outcomes**Frequency of participants reported never (1) to always (5) for the frequency of (A) avoiding crowds, (B) washing hands and (C) wearing a mask. Numbers of reported contacts for (D) physical, (E) non-physical without 1m distancing, and (F) non-physical with 1m distancing are shown.


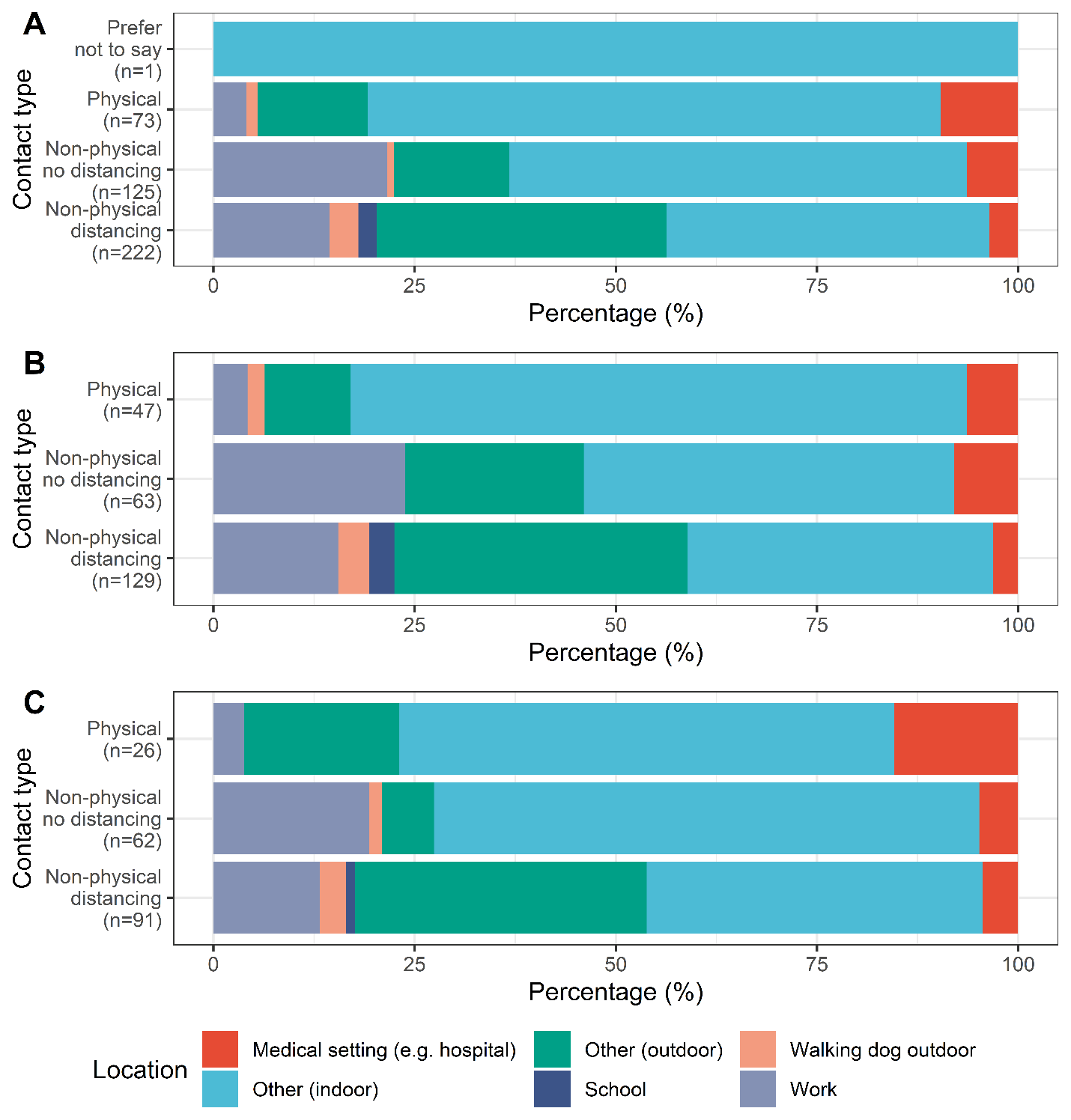
**Figure S4. Locations of contact for each contact type**Proportions of each location in which contacts occurred are shown for (A) all participants, (B) participants with Chance score equal to or lower than the median Chance score of the sample, and (C) participants with the score higher than the median value. One contact that was categorised as ‘prefer not to say’ was removed from (C).

Table S1. Summary of demographic and socio-economic status of the study population.

| **Variable** |  | **n** | **Crowds^1^** | **Hands^1^** | **Masks^1^** | **Contacts^2^** |
| --- | --- | --- | --- | --- | --- | --- |
| Age | Under 25 | 19 | 15.6% | 63.2% | 26.3% | 9.06 |
| (n = 231) | 25-34 | 88 | 13.6% | 79.5% | 26.1% | 6.04 |
|  | 35-44 | 48 | 16.7% | 83.3% | 27.1% | 3.36 |
|  | 45-54 | 27 | 22.2% | 74.1% | 25.9% | 7.17 |
|  | 55-64 | 35 | 34.3% | 80% | 25.7% | 5.30 |
|  | 65+ | 14 | 42.9% | 85.7% | 21.4% | 4.67 |
|  |  |  |  |  |  |  |
| Gender | Male | 62 | 9.7% | 67.7% | 22.6% | 6.46 |
| (n = 231) | Female | 167 | 24.6% | 82.6% | 26.3% | 5.41 |
|  | Other | 2 | 0% | 100% | 100% | 2.5 |
|  |  |  |  |  |  |  |
| Ethnicity | White - British | 181 | 21.5% | 77.9% | 25.4% | 5.48 |
| (n = 226) | White - Other | 28 | 21.4% | 85.7% | 28.6% | 6.79 |
|  | Other | 17 | 11.8% | 88.2% | 23.5% | 5.38 |
|  |  |  |  |  |  |  |
| Marital status | Married | 135 | 22.2% | 77.0% | 25.9% | 5.57 |
| (n = 225) | Separated | 13 | 38.5% | 92.3% | 30.8% | 3.83 |
|  | Never married | 77 | 15.6% | 81.8% | 23.4% | 6.36 |
|  |  |  |  |  |  |  |
| Education | Postgraduate | 122 | 24.6% | 82.0% | 26.2% | 4.32 |
| (n = 231) | A level/Higher | 10 | 30% | 80.0% | 50.0% | 9.33 |
|  | Further education | 19 | 15.8% | 84.2% | 26.3% | 4.6 |
|  | GCSE/O-levels | 8 | 12.5% | 100.0% | 37.5% | 2.71 |
|  | University (first) degree | 69 | 13.0% | 69.6% | 18.8% | 8 |
|  | Prefer not to say | 3 | 33.3% | 66.7% | 66.7% | 6 |
|  |  |  |  |  |  |  |
| Income | <24400 | 49 | 18.4% | 83.7% | 22.4% | 7.48 |
| (n = 229) | <33600 | 43 | 25.6% | 76.7% | 25.6% | 6.05 |
|  | <41100 | 47 | 19.1% | 76.6% | 21.3% | 5.09 |
|  | Above 41100 | 57 | 14.0% | 75.4% | 21.1% | 5.81 |
|  | Prefer not to answer | 33 | 27.3% | 84.8% | 45.5% | 3.56 |
|  |  |  |  |  |  |  |
| Location | England | 175 | 18.9% | 80.6% | 25.7% | 6.05 |
| (n = 232) | Northern Ireland | 1 | 0.0% | 100.0% | 100.0% | 4 |
|  | Scotland | 29 | 20.7% | 62.1% | 31.0% | 1.92 |
|  | Wales | 21 | 23.8% | 81.0% | 19.0% | 8.21 |
|  | Unknown UK | 6 | 60.0% | 100% | 20.0% | 0 |
|  |  |  |  |  |  |  |
| Key worker | No | 147 | 22.4% | 82.3% | 27.9% | 4.71 |
| (n = 232) | Yes - Health or social care | 45 | 8.9% | 68.9% | 11.1% | 7.88 |
|  | Yes - Other key worker | 35 | 22.9% | 74.3% | 37.1% | 7.64 |
|  | Other | 5 | 50.0% | 100% | 25% | 1 |
|  |  |  |  |  |  |  |
| Employment | Full time | 145 | 16.6% | 78.6% | 25.5% | 5.9 |
| (n = 227) | Not seek work | 5 | 20% | 80% | 40% | 2 |
|  | Part work | 30 | 16.7% | 66.7% | 16.7% | 8.04 |
|  | Retired | 21 | 52.4% | 85.7% | 19.0% | 5.88 |
|  | Studying | 19 | 21.1% | 84.2% | 31.6% | 3.06 |
|  | Unemployed and seeking work | 7 | 28.6% | 100% | 57.1% | 3.71 |
|  |  |  |  |  |  |  |
| Work status | Work from home | 97 | 23.7% | 79.4% | 25.8% | 2.42 |
| (n = 228) | Work as usual | 66 | 4.5% | 78.8% | 22.7% | 11.4 |
|  | Laidoff | 23 | 21.7% | 82.6% | 30.4% | 4.35 |
|  | Other | 42 | 35.7% | 73.8% | 23.8% | 5.46 |
|  |  |  |  |  |  |  |

**^1^** Percentage of individuals who always implement the behaviour of interest: avoiding crowds, washing hands and wearing masks.  **^2^** Mean number of overall contacts that are not adjusted by the sampling weight**.**

Table S2. Summary of variables related to Covid-19 in the study population

| **Variable** |  | **n** | **%** |
| --- | --- | --- | --- |
| Believe already have been infected with Covid-19 | Yes | 38 | 16.7% |
| (n = 228) | No | 50 | 21.9% |
|  | Don't know | 140 | 61.4% |
|  |  |  |  |
| Received Covid-19 test | Yes | 53 | 23.1% |
| (n = 229) | No | 176 | 76.9% |
|  |  |  |  |
| Type of test received | PCR | 31 | 58.4% |
| (n = 53) | Antibody | 12 | 22.6% |
|  | Both | 10 | 19.0% |
|  |  |  |  |
| PCR result | Positive | 1^1^ | 2.4% |
| (n = 40) | Negative | 39 | 95.1% |
|  |  |  |  |
| Antibody test result | Positive | 3 | 13.6% |
| (n = 22) | Negative | 18 | 81.8% |
|  | Don't know | 1 | 4.5% |
|  |  |  |  |
| Number of housemates received Covid-19 test | 0 | 141 | 72.7% |
| (n = 194) | 1 | 41 | 21.1% |
|  | 2 | 8 | 4.1% |
|  | 3 | 2 | 1.5% |
|  | 4 | 1 | 0.5% |
|  | Don't know | 1 | 0.5% |
|  |  |  |  |
| Number of housemates tested positive for Covid-19 test | 0 | 42 | 80.8% |
| (n = 52) | 1 | 6 | 11.5% |
|  | 2 | 1 | 1.9% |
|  | Don't know | 3 | 5.8% |
|  |  |  |  |
| Current symptom^2^ |  |  |  |
| (n = 232) | Cough | 4 | 1.7% |
|  | Blocked nose | 15 | 6.5% |
|  | Unusually tired | 23 | 9.9% |
|  | Sore throat | 15 | 6.5% |
|  | Shortness of breath | 7 | 3.0% |
|  | Fever | 0 | 0.0% |
|  | Aches | 18 | 7.8% |
|  | Loss of smell | 1 | 0.4% |
|  | No symptom | 165 | 71.1% |
|  |  |  |  |
| Trust in Covid-19 test | 1 (Don't trust at all) | 4 | 2.5% |
| (n = 159) | 2 | 4 | 2.5% |
|  | 3 | 25 | 15.7% |
|  | 4 | 62 | 40.0% |
|  | 5 (Strongly trust) | 64 | 40.3% |
|  |  |  |  |
| Likelihood of Covid-19 infection in the next 4 weeks | 1 (Not likely at all) | 39 | 17.1% |
| (n = 228) | 2 | 62 | 27.2% |
|  | 3 | 55 | 24.1% |
|  | 4 | 23 | 10.1% |
|  | 5 | 25 | 11.0% |
|  | 6 | 14 | 6.1% |
|  | 7 | 6 | 2.6% |
|  | 8 | 3 | 1.3% |
|  | 9 | 1 | 0.4% |
|  | 10 (Extremely likely) | 0 | 0.0% |

^1^ This individual also tested positive in the antibody test, making a total of three individuals tested
positive in our dataset
^2^ Participants could choose all that applied

**4.2. Principal component analysis for trust dimensions**

Six observations were removed from this analysis due to missing data in at least one of the trust variables. The first two principal component variables (PC) had eigenvalue greater than 1, hence two components were extracted and subjected to varimax rotation. The first PC (PC1) explained the majority of the variance (52.4%), and loaded onto the majority of items (Table S3). This component is interpreted as general trust towards the government. The second PC (PC2) explained 8.1% of the variance, which was dominated by one ‘Integrity’ and one ‘Care’ item. This component is interpreted as the government’s interest in public perceived by the participants. PC1 was statistically associated with Internal (r = 0.21, p = 0.001) and Powerful others (r = 0.18, p =0.006) HLC scores, but not with Chance score (r = 0.06, p = 0.37). PC2 had no statistical associations with any of HLC scores.

Table S3. The first two Principal components (PCs) and their loadings on 15 trust and value similarity items

| **Concept** | **Item** | **PC 1** | **PC 2** |
| --- | --- | --- | --- |
| Competence | The Government is doing a good job in relation to COVID-19 | 0.85 | -0.14 |
| Competence | The Government has been organising its response to COVID-19 competently | 0.83 | -0.19 |
| Competence | The Government has had the necessary skilled people to manage COVID-19 | 0.68 | 0.03 |
| Credibility | The Government has distorted facts about COVID-19 to make its case for its policy | -0.61 | 0.32 |
| Credibility | The Government has ignored the views of scientists who disagree with them about COVID-19 | -0.68 | 0.34 |
| Integrity | The Government has been too influenced by public opinion regarding COVID-19 | -0.20 | 0.78 |
| Integrity | The Government has acknowledged mistakes it has made about COVID-19 | 0.69 | -0.05 |
| Reliability | The Government has taken its commitments to reducing COVID-19 seriously | 0.83 | 0.02 |
| Reliability | We cannot rely on the Government to ensure that COVID-19 is managed properly | -0.71 | 0.15 |
| Openness | The Government has been open and honest about COVID-19 | 0.80 | 0.08 |
| Care | The Government has been interested in what the general public thinks about COVID-19 | 0.50 | 0.58 |
| Care | The Government cares about reducing COVID-19 | 0.73 | 0.11 |
| Fairness | The Government has considered all the arguments for and against its measures to deal with COVID-19 | 0.81 | 0.01 |
| Fairness | Decisions made by the Government about COVID-19 have been fair and just | 0.83 | 0.02 |
| Value similarity | The Government has the same opinion as me about the best way to control COVID-19 | 0.79 | -0.07 |

**4.3 Health behaviours**

Chance score was negatively associated with all the health behaviour variables both in the univariable and the multivariable analysis; the adjusted odds ratio (aOR) was 0.46 (95% confidence interval (CI) 0.25 – 0.86) for avoiding crowds, 0.35 (95%CI 0.17 – 0.70) for washing hands, and 0.58 (95%CI 0.34 – 0.99) for wearing masks (Table S4 and see Supplementary Tables S6 – S8 for the full model). No interactions were identified between Chance score and other confounding variables. Sensitivity analyses showed that the change in the cut-off and the exclusion of responses after 8^th^ August would result in a quantitative change in the coefficient of Chance score but generally the negative associations remained (Table S6 – S8).

For other health locus of control (HLC) variables other than Chance, Powerful others variable remained in the final model for washing hands (aOR 1.86, 95%CI 1.05 – 3.28, p = 0.03) and wearing masks (aOR 1.70, 95%CI 0.98 – 2.94, p = 0.05). No associations were identified between Internal score and outcomes. Trust variables did not remain in the final models except for wearing masks; a unit increase in PC1 score decreased the probability of wearing masks (aOR 0.63, 95%CI 0.43 – 0.93, p = 0.02).

Table S4. The effect of Chance variable on the three health behaviour variables derived from the final multivariable logistic regression models

| **Outcome variable** | **aOR**^1^ | **2.5%** | **97.5%** | **p value** |
| --- | --- | --- | --- | --- |
| Avoiding crowds^2^ | 0.46 | 0.25 | 0.86 | 0.02 |
| Washing hands^3^ | 0.35 | 0.17 | 0.70 | 0.003 |
| Wearing mask^4^ | 0.58 | 0.34 | 0.99 | 0.046 |

^1^ Adjusted odds ratio ^2^ The final model included Age, Gender, Work status and live with 5 – 18 years old individual.
^3^ The final model included Powerful others variable, Age, Gender, Key worker, Work status, Employment status, live with y years old or younger, live with 5 – 18 years old individual, and currently have shortness of breath.
^4^ The final model included Powerful others variable, PC1, Age, Gender, Key worker and Income.

Table S5a. Univariable analysis of regression modelling for preventive behaviours

|  |  | **Avoid crowds** | | | |  | **Wash hands** | | | |  | **Wear masks** | | | |  |
| --- | --- | --- | --- | --- | --- | --- | --- | --- | --- | --- | --- | --- | --- | --- | --- | --- |
| Variable | Level | Coefficient (log odds) | SE | P value | P value^1^ |  | Coefficient (log odds) | SE | P value | P value^1^ |  | Coefficient (log odds) | SE | P value | P value^1^ |  |
| PC1 |  | 0.16 | 0.17 | 0.33 |  |  | -0.16 | 0.16 | 0.34 |  |  | -0.41 | 0.17 | **0.01** |  |  |
| PC2 |  | 0.08 | 0.17 | 0.62 |  |  | 0.19 | 0.17 | 0.24 |  |  | -0.12 | 0.15 | 0.43 |  |  |
|  |  |  |  |  |  |  |  |  |  |  |  |  |  |  |  |  |
| Chance |  | -0.49 | 0.26 | **0.06** |  |  | -0.42 | 0.25 | **0.09** |  |  | -0.59 | 0.25 | **0.02** |  |  |
| Internal |  | 0.17 | 0.24 | 0.47 |  |  | 0.20 | 0.23 | 0.39 |  |  | 0.02 | 0.22 | 0.94 |  |  |
| External |  | 0.11 | 0.24 | 0.64 |  |  | 0.59 | 0.23 | **0.01** |  |  | 0.23 | 0.22 | 0.30 |  |  |
|  |  |  |  |  |  |  |  |  |  |  |  |  |  |  |  |  |
| Age | 25-34 |  |  |  | **0.06** |  |  |  |  | 0.99 |  |  |  |  | 1.00 |  |
|  | 35-44 | 0.24 | 0.50 | 0.63 |  |  | 0.25 | 0.47 | 0.59 |  |  | 0.05 | 0.41 | 0.91 |  |  |
|  | 45-54 | 0.59 | 0.56 | 0.29 |  |  | -0.31 | 0.51 | 0.55 |  |  | -0.01 | 0.50 | 0.98 |  |  |
|  | 55-64 | 1.20 | 0.47 | 0.01 |  |  | 0.03 | 0.50 | 0.96 |  |  | -0.02 | 0.46 | 0.96 |  |  |
|  | 65+ | 1.56 | 0.62 | 0.01 |  |  | 0.43 | 0.81 | 0.59 |  |  | -0.26 | 0.70 | 0.71 |  |  |
|  | Under 25 | 0.17 | 0.70 | 0.81 |  |  | -0.82 | 0.54 | 0.13 |  |  | 0.01 | 0.57 | 0.99 |  |  |
|  |  |  |  |  |  |  |  |  |  |  |  |  |  |  |  |  |
| Gender | Female |  |  |  | **0.02** |  |  |  |  | 0.38 |  |  |  |  | **0.06** |  |
|  | Male | -1.11 | 0.47 | 0.02 |  |  | -0.82 | 0.34 | 0.02 |  |  | -0.20 | 0.35 | 0.56 |  |  |
|  | Other | -14.44 | 1029.12 | 0.99 |  |  | 14.01 | 1029.12 | 0.99 |  |  | 16.59 | 1029.12 | 0.99 |  |  |
|  |  |  |  |  |  |  |  |  |  |  |  |  |  |  |  |  |
| Ethnicity | White - British |  |  |  | 0.60 |  |  |  |  | 1.00 |  |  |  |  | 0.92 |  |
|  | White - Other | -0.01 | 0.49 | 0.99 |  |  | 0.08 | 0.08 | 0.34 |  |  | 0.16 | 0.45 | 0.72 |  |  |
|  | Other | -0.72 | 0.77 | 0.35 |  |  | 0.10 | 0.10 | 0.31 |  |  | -0.10 | 0.60 | 0.86 |  |  |
|  |  |  |  |  |  |  |  |  |  |  |  |  |  |  |  |  |
| Marital status | Married |  |  |  | **0.16** |  |  |  |  | 0.31 |  |  |  |  | 0.83 |  |
|  | Separated | 0.78 | 0.61 | 0.20 |  |  | 1.27 | 1.06 | 0.23 |  |  | 0.24 | 0.63 | 0.71 |  |  |
|  | Never married | -0.44 | 0.38 | 0.25 |  |  | 0.29 | 0.36 | 0.41 |  |  | -0.14 | 0.33 | 0.68 |  |  |
|  |  |  |  |  |  |  |  |  |  |  |  |  |  |  |  |  |
| Education | Post graduate |  |  |  | 0.39 |  |  |  |  | **0.14** |  |  |  |  | 0.20 |  |
|  | A level/Higher | 0.27 | 0.72 | 0.70 |  |  | -0.13 | 0.82 | 0.88 |  |  | 1.03 | 0.67 | 0.12 |  |  |
|  | Further education | -0.55 | 0.66 | 0.40 |  |  | 0.16 | 0.67 | 0.81 |  |  | 0.00 | 0.56 | 0.99 |  |  |
|  | GCSE/O-levels | -0.83 | 1.09 | 0.45 |  |  | 15.05 | 848.37 | 0.99 |  |  | 0.52 | 0.76 | 0.49 |  |  |
|  | Prefer not to say | 0.43 | 1.24 | 0.73 |  |  | -0.82 | 1.25 | 0.51 |  |  | 1.73 | 1.24 | 0.16 |  |  |
|  | University (first) degree | -0.78 | 0.41 | 0.06 |  |  | -0.69 | 0.35 | 0.05 |  |  | -0.43 | 0.37 | 0.25 |  |  |
|  |  |  |  |  |  |  |  |  |  |  |  |  |  |  |  |  |
| Income | <24400 |  |  |  | 0.53 |  |  |  |  | 0.71 |  |  |  |  | **0.16** |  |
|  | <33600 | 0.42 | 0.51 | 0.40 |  |  | -0.44 | 0.53 | 0.41 |  |  | 0.17 | 0.49 | 0.85 |  |  |
|  | <41100 | 0.05 | 0.52 | 0.92 |  |  | -0.45 | 0.52 | 0.39 |  |  | -0.07 | 0.49 | 0.50 |  |  |
|  | Above 41100 | -0.32 | 0.53 | 0.55 |  |  | -0.51 | 0.49 | 0.30 |  |  | -0.08 | 0.47 | 0.69 |  |  |
|  | Prefer not to answer | 0.51 | 0.54 | 0.34 |  |  | 0.09 | 0.62 | 0.89 |  |  | 1.06 | 0.49 | 0.11 |  |  |
|  |  |  |  |  |  |  |  |  |  |  |  |  |  |  |  |  |
| Location | England |  |  |  | 0.33 |  |  |  |  | **0.11** |  |  |  |  | 0.44 |  |
|  | Northern Ireland | -13.11 | 882.74 | 0.99 |  |  | 15.14 | 2399.54 | 1.00 |  |  | 15.63 | 882.74 | 0.99 |  |  |
|  | Scotland | 0.12 | 0.50 | 0.82 |  |  | -0.93 | 0.43 | 0.03 |  |  | 0.26 | 0.44 | 0.55 |  |  |
|  | Wales | 0.30 | 0.55 | 0.59 |  |  | 0.02 | 0.59 | 0.97 |  |  | -0.39 | 0.58 | 0.51 |  |  |
|  | Unknown UK | 1.86 | 0.93 | 0.05 |  |  | 15.14 | 1073.11 | 0.99 |  |  | -0.33 | 1.13 | 0.77 |  |  |
|  |  |  |  |  |  |  |  |  |  |  |  |  |  |  |  |  |
| Key worker | No |  |  |  | **0.08** |  |  |  |  | **0.12** |  |  |  |  | **0.04** |  |
|  | Yes - Health care | -1.09 | 0.56 | 0.05 | . |  | -0.74 | 0.39 | 0.06 |  |  | -1.13 | 0.51 | 0.03 |  |  |
|  | Yes - Other key worker | 0.02 | 0.45 | 0.96 |  |  | -0.48 | 0.44 | 0.28 |  |  | 0.42 | 0.40 | 0.28 |  |  |
|  | Other | 1.24 | 1.02 | 0.22 |  |  | 15.03 | 1199.77 | 0.99 |  |  | -0.15 | 1.17 | 0.90 |  |  |
|  |  |  |  |  |  |  |  |  |  |  |  |  |  |  |  |  |
| Employment | Full time |  |  |  | **0.03** |  |  |  |  | 0.24 |  |  |  |  | 0.32 |  |
|  | Not seek work | 0.23 | 1.14 | 0.84 |  |  | 0.08 | 1.14 | 0.94 |  |  | 0.67 | 0.93 | 0.48 |  |  |
|  | Part work | 0.01 | 0.54 | 0.99 |  |  | -0.61 | 0.44 | 0.16 |  |  | -0.54 | 0.53 | 0.31 |  |  |
|  | Retired | 1.71 | 0.49 | 0.00 |  |  | 0.49 | 0.66 | 0.46 |  |  | -0.38 | 0.59 | 0.52 |  |  |
|  | Studying | 0.30 | 0.61 | 0.62 |  |  | 0.37 | 0.66 | 0.57 |  |  | 0.30 | 0.53 | 0.57 |  |  |
|  | Unemployed and seeking work | 0.70 | 0.87 | 0.42 |  |  | 15.26 | 906.94 | 0.99 |  |  | 1.36 | 0.79 | 0.08 |  |  |
|  |  |  |  |  |  |  |  |  |  |  |  |  |  |  |  |  |
| Work status | Work from home |  |  |  | **0.00** |  |  |  |  | 0.85 |  |  |  |  | 0.90 |  |
|  | Work as usual | -1.88 | 0.64 | 0.00 |  |  | -0.04 | 0.39 | 0.93 |  |  | -0.17 | 0.37 | 0.66 |  |  |
|  | Laidoff | -0.11 | 0.56 | 0.84 |  |  | 0.21 | 0.60 | 0.73 |  |  | 0.23 | 0.51 | 0.65 |  |  |
|  | Other | 0.58 | 0.40 | 0.15 |  |  | -0.31 | 0.43 | 0.47 |  |  | -0.11 | 0.43 | 0.81 |  |  |
|  |  |  |  |  |  |  |  |  |  |  |  |  |  |  |  |  |
| Live with others | No |  |  |  |  |  |  |  |  |  |  |  |  |  |  |  |
|  | Yes | -0.36 | 0.45 | 0.42 |  |  | -0.67 | 0.56 | 0.23 |  |  | 0.43 | 0.48 | 0.37 |  |  |
|  |  |  |  |  |  |  |  |  |  |  |  |  |  |  |  |  |
| Live with 4y or younger | No |  |  |  |  |  |  |  |  |  |  |  |  |  |  |  |
|  | Yes | -0.25 | 0.66 | 0.70 |  |  | -0.66 | 0.53 | 0.21 |  |  | 0.11 | 0.55 | 0.85 |  |  |
|  |  |  |  |  |  |  |  |  |  |  |  |  |  |  |  |  |
| Live with 5y - 18y | No |  |  |  |  |  |  |  |  |  |  |  |  |  |  |  |
|  | Yes | 0.48 | 0.45 | 0.29 |  |  | 0.96 | 0.63 | **0.13** |  |  | 0.29 | 0.43 | 0.50 |  |  |
|  |  |  |  |  |  |  |  |  |  |  |  |  |  |  |  |  |
| Live with 19 - 64 y | No |  |  |  |  |  |  |  |  |  |  |  |  |  |  |  |
|  | Yes | -0.47 | 0.38 | 0.21 |  |  | -0.61 | 0.44 | **0.17** |  |  | -0.17 | 0.36 | 0.63 |  |  |
|  |  |  |  |  |  |  |  |  |  |  |  |  |  |  |  |  |
| Live with 65y or greater | No |  |  |  |  |  |  |  |  |  |  |  |  |  |  |  |
|  | Yes | 0.75 | 0.53 | **0.16** |  |  | 0.84 | 0.77 | 0.28 |  |  | 0.11 | 0.55 | 0.85 |  |  |
|  |  |  |  |  |  |  |  |  |  |  |  |  |  |  |  |  |
| Guardian of children under 18 y | No |  |  |  |  |  |  |  |  |  |  |  |  |  |  |  |
|  | Yes | -0.24 | 0.45 | 0.60 |  |  | 0.10 | 0.43 | 0.81 |  |  | 0.26 | 0.38 | 0.50 |  |  |
|  |  |  |  |  |  |  |  |  |  |  |  |  |  |  |  |  |
| Think I have been already infected | No |  |  |  | **0.07** |  |  |  |  | 0.45 |  |  |  |  | **0.20** |  |
|  | Yes | -1.28 | 0.63 | 0.04 |  |  | -0.53 | 0.42 | 0.20 |  |  | -0.69 | 0.48 | 0.16 |  |  |
|  | Don't know | -0.21 | 0.41 | 0.60 |  |  | -0.05 | 0.41 | 0.91 |  |  | 0.23 | 0.36 | 0.51 |  |  |
|  |  |  |  |  |  |  |  |  |  |  |  |  |  |  |  |  |
| Received Covid-19 test | No |  |  |  |  |  |  |  |  |  |  |  |  |  |  |  |
|  | Yes | -0.26 | 0.41 | 0.52 |  |  | 0.72 | 0.44 | 0.10 |  |  | -0.11 | 0.36 | 0.75 |  |  |
|  |  |  |  |  |  |  |  |  |  |  |  |  |  |  |  |  |
| I tested positive for Covid-19 | No |  |  |  | 0.44 |  |  |  |  | **0.13** |  |  |  |  | 0.40 |  |
|  | Yes | -14.03 | 840.27 | 0.99 |  |  | 13.73 | 840.27 | 0.99 |  |  | -14.49 | 840.27 | 0.99 |  |  |
|  | Not applicable (not received test) | 0.22 | 0.41 | 0.60 |  |  | -0.68 | 0.44 | 0.13 |  |  | 0.06 | 0.36 | 0.86 |  |  |
|  |  |  |  |  |  |  |  |  |  |  |  |  |  |  |  |  |
| Housemate received Covid-19 test | No |  |  |  | 0.32 |  |  |  |  | 0.38 |  |  |  |  | 0.44 |  |
|  | Yes | -0.59 | 0.45 | 0.19 |  |  | 0.56 | 0.43 | 0.20 |  |  | 0.39 | 0.35 | 0.28 |  |  |
|  | Not applicable (living alone) | 0.13 | 0.43 | 0.76 |  |  | 0.31 | 0.46 | 0.51 |  |  | -0.18 | 0.44 | 0.69 |  |  |
|  |  |  |  |  |  |  |  |  |  |  |  |  |  |  |  |  |
| Housemate tested positive for Covid-19 | No |  |  |  | **0.02** |  |  |  |  | 0.28 |  |  |  |  | 0.44 |  |
|  | Yes | -15.01 | 906.94 | 0.99 |  |  | -0.94 | 0.79 | 0.23 |  |  | 0.73 | 0.79 | 0.35 |  |  |
|  | Not applicable | 0.75 | 0.34 | 0.03 |  |  | 0.36 | 0.38 | 0.35 |  |  | -0.27 | 0.35 | 0.44 |  |  |
|  |  |  |  |  |  |  |  |  |  |  |  |  |  |  |  |  |
| Currently have symptoms- cough | No |  |  |  |  |  |  |  |  |  |  |  |  |  |  |  |
|  | Yes | -15.22 | 1199.77 | 0.99 |  |  | -1.34 | 1.01 | **0.19** |  |  | -14.54 | 727.70 | 0.98 |  |  |
|  |  |  |  |  |  |  |  |  |  |  |  |  |  |  |  |  |
| Currently have symptoms- blocked nose | No |  |  |  |  |  |  |  |  |  |  |  |  |  |  |  |
|  | Yes | 0.73 | 0.57 | 0.21 |  |  | 0.08 | 0.67 | 0.91 |  |  | 0.99 | 0.54 | **0.07** |  |  |
|  |  |  |  |  |  |  |  |  |  |  |  |  |  |  |  |  |
| Currently have symptoms- feeling unusually tired | No |  |  |  |  |  |  |  |  |  |  |  |  |  |  |  |
|  | Yes | 0.61 | 0.49 | 0.21 |  |  | -0.03 | 0.53 | 0.95 |  |  | 0.25 | 0.48 | 0.61 |  |  |
|  |  |  |  |  |  |  |  |  |  |  |  |  |  |  |  |  |
| Currently have symptoms- sore throat | No |  |  |  |  |  |  |  |  |  |  |  |  |  |  |  |
|  | Yes | 0.38 | 0.61 | 0.53 |  |  | 0.08 | 0.67 | 0.91 |  |  | 0.69 | 0.55 | 0.21 |  |  |
|  |  |  |  |  |  |  |  |  |  |  |  |  |  |  |  |  |
| Currently have symptoms- shortness of breath | No |  |  |  |  |  |  |  |  |  |  |  |  |  |  |  |
|  | Yes | -15.24 | 906.94 | 0.99 |  |  | -1.07 | 0.78 | **0.17** |  |  | -15.56 | 906.94 | 0.99 |  |  |
|  |  |  |  |  |  |  |  |  |  |  |  |  |  |  |  |  |
| Currently have symptoms- aches and pains | No |  |  |  |  |  |  |  |  |  |  |  |  |  |  |  |
|  | Yes | 0.45 | 0.55 | 0.42 |  |  | 0.82 | 0.77 | 0.29 |  |  | -0.22 | 0.59 | 0.70 |  |  |
|  |  |  |  |  |  |  |  |  |  |  |  |  |  |  |  |  |
| Currently have symptoms- loss of sense of smell | No |  |  |  |  |  |  |  |  |  |  |  |  |  |  |  |
|  | Yes | -13.21 | 882.74 | 0.99 |  |  | 13.26 | 882.74 | 0.99 |  |  | -13.52 | 882.74 | 0.99 |  |  |
|  |  |  |  |  |  |  |  |  |  |  |  |  |  |  |  |  |
| Currently have at least one of symptoms above | Yes |  |  |  |  |  |  |  |  |  |  |  |  |  |  |  |
|  | No (no symptoms) | -0.20 | 0.35 | 0.57 |  |  | -0.40 | 0.38 | 0.29 |  |  | -0.41 | 0.32 | 0.20 |  |  |
|  |  |  |  |  |  |  |  |  |  |  |  |  |  |  |  |  |
| Trust test results for Covid-19 | | 0.13 | 0.25 | 0.61 |  |  | -0.02 | 0.20 | 0.91 |  |  | -0.03 | 0.19 | 0.86 |  | |
|  |  |  |  |  |  |  |  |  |  |  |  |  |  |  |  |  |
| Likely to be infected with Covid-19 in the next 4 weeks |  | -0.05 | 0.10 | 0.63 |  |  | 0.08 | 0.10 | 0.44 |  |  | -0.02 | 0.09 | 0.78 |  |  |

^1^ p value from log-likelihood ratio test

Table S5b. Univariable analysis of regression modelling for contacts

|  |  | **Physical contact** | | | |  | **Non-physical within 1m** | | | |  | **Non-physical >1m** | | | |
| --- | --- | --- | --- | --- | --- | --- | --- | --- | --- | --- | --- | --- | --- | --- | --- |
| Variable | Level | Coefficient (log count) | SE | P value | P value ^1^ | | Coefficient (log count) | SE | P value | P value^1^ |  | Coefficient (log count) | SE | P value | P value ^1^ |
| PC1 |  | -0.07 | 0.10 | 0.47 |  |  | 0.09 | 0.05 | **0.11** |  |  | -0.05 | 0.04 | 0.17 |  |
| PC2 |  | 0.06 | 0.09 | 0.53 |  |  | 0.05 | 0.05 | 0.38 |  |  | 0.11 | 0.04 | **<0.001** |  |
|  |  |  |  |  |  |  |  |  |  |  |  |  |  |  |  |
| Chance |  | 0.10 | 0.14 | 0.46 |  |  | 0.53 | 0.08 | **<0.001** |  |  | 0.33 | 0.06 | **<0.001** |  |
| Internal |  | 0.13 | 0.14 | 0.33 |  |  | -0.14 | 0.07 | **0.05** |  |  | 0.02 | 0.05 | 0.69 |  |
| External |  | -0.31 | 0.12 | **0.01** |  |  | -0.28 | 0.07 | **<0.001** |  |  | -0.22 | 0.05 | **<0.001** |  |
|  |  |  |  |  |  |  |  |  |  |  |  |  |  |  |  |
| Age | 25-34 | Base |  |  | **<0.001** |  | Base |  |  | **<0.001** | | Base |  |  | **<0.001** |
|  | 35-44 | -0.80 | 0.35 | 0.02 |  |  | -1.12 | 0.18 | 0.00 |  |  | -0.30 | 0.12 | 0.01 |  |
|  | 45-54 | 0.25 | 0.30 | 0.40 |  |  | -0.96 | 0.22 | 0.00 |  |  | 0.54 | 0.11 | 0.00 |  |
|  | 55-64 | 0.33 | 0.28 | 0.24 |  |  | -0.72 | 0.19 | 0.00 |  |  | 0.29 | 0.11 | 0.01 |  |
|  | 65+ | 0.43 | 0.41 | 0.29 |  |  | -0.32 | 0.23 | 0.17 |  |  | -0.07 | 0.18 | 0.69 |  |
|  | Under 25 | 1.18 | 0.25 | 0.00 |  |  | 0.03 | 0.16 | 0.84 |  |  | 0.37 | 0.12 | 0.00 |  |
|  |  |  |  |  |  |  |  |  |  |  |  |  |  |  |  |
| Gender | Female | Base |  |  | 0.57 |  | Base |  |  | **<0.001** | | Base |  |  | 0.14 |
|  | Male | -0.22 | 0.22 | 0.31 |  |  | 0.50 | 0.11 | 0.00 |  |  | 0.04 | 0.08 | 0.59 |  |
|  | Other | -0.24 | 1.01 | 0.81 |  |  | -1.16 | 1.00 | 0.25 |  |  | -0.92 | 0.58 | 0.11 |  |
|  |  |  |  |  |  |  |  |  |  |  |  |  |  |  |  |
| Ethnicity | White - British | Base |  |  | 0.37 |  | Base |  |  | **0.01** |  | Base |  |  | **<0.001** |
|  | White - Other | -0.42 | 0.32 | 0.18 |  |  | 0.12 | 0.15 | 0.43 |  |  | 0.22 | 0.10 | 0.04 |  |
|  | Other | -0.12 | 0.37 | 0.74 |  |  | 0.51 | 0.16 | 0.00 |  |  | -0.56 | 0.18 | 0.00 |  |
|  |  |  |  |  |  |  |  |  |  |  |  |  |  |  |  |
| Marital status | Married | Base |  |  | 0.67 |  | Base |  |  | **<0.001** | | Base |  |  | **0.10** |
|  | Separated | 0.25 | 0.37 | 0.50 |  |  | -1.21 | 0.38 | 0.00 |  |  | -0.35 | 0.18 | 0.05 |  |
|  | Never married | 0.13 | 0.20 | 0.51 |  |  | 0.24 | 0.11 | 0.03 |  |  | -0.08 | 0.08 | 0.32 |  |
|  |  |  |  |  |  |  |  |  |  |  |  |  |  |  |  |
| Education | Post graduate | Base |  |  | **<0.001** |  | Base |  |  | **<0.001** | | Base |  |  | **<0.001** |
|  | A level/Higher | 1.18 | 0.35 | 0.00 |  |  | 1.44 | 0.18 | 0.00 |  |  | 0.34 | 0.17 | 0.04 |  |
|  | Further education | 0.11 | 0.44 | 0.80 |  |  | 0.05 | 0.25 | 0.83 |  |  | 0.24 | 0.15 | 0.11 |  |
|  | GCSE/O-levels | 0.55 | 0.52 | 0.29 |  |  | -0.06 | 0.36 | 0.86 |  |  | -0.77 | 0.32 | 0.02 |  |
|  | Prefer not to say | 0.27 | 1.01 | 0.79 |  |  | 0.50 | 0.71 | 0.49 |  |  | 1.18 | 0.32 | 0.00 |  |
|  | University (first) degree | 0.88 | 0.20 | 0.00 |  |  | 0.82 | 0.12 | 0.00 |  |  | 0.51 | 0.08 | 0.00 |  |
|  |  |  |  |  |  |  |  |  |  |  |  |  |  |  |  |
| Income | <24400 | Base |  |  | 0.30 |  | Base |  |  | **<0.001** | | Base |  |  | **<0.001** |
|  | <33600 | 0.05 | 0.27 | 0.85 |  |  | -0.08 | 0.15 | 0.59 |  |  | -0.23 | 0.11 | 0.04 |  |
|  | <41100 | -0.38 | 0.28 | 0.18 |  |  | -0.53 | 0.16 | 0.00 |  |  | -0.36 | 0.11 | 0.00 |  |
|  | Above 41100 | -0.31 | 0.27 | 0.25 |  |  | -0.17 | 0.14 | 0.23 |  |  | -0.25 | 0.10 | 0.02 |  |
|  | Prefer not to answer | -0.05 | 0.30 | 0.88 |  |  | -0.85 | 0.22 | 0.00 |  |  | -0.49 | 0.14 | 0.00 |  |
|  |  |  |  |  |  |  |  |  |  |  |  |  |  |  |  |
| Location | England | Base |  |  | **0.03** |  | Base |  |  | **<0.001** | | Base |  |  | **<0.001** |
|  | Northern Ireland | -13.87 | 773.78 | 0.99 |  |  | 0.68 | 0.50 | 0.18 |  |  | -14.66 | 469.32 | 0.98 |  |
|  | Scotland | -1.09 | 0.46 | 0.02 |  |  | -1.20 | 0.27 | 0.00 |  |  | -0.94 | 0.17 | 0.00 |  |
|  | Wales | 0.18 | 0.29 | 0.52 |  |  | 0.07 | 0.17 | 0.68 |  |  | 0.54 | 0.10 | 0.00 |  |
|  | Unknown UK | -0.95 | 1.01 | 0.34 |  |  | -15.01 | 547.15 | 0.98 |  |  | -1.36 | 0.58 | 0.02 |  |
|  |  |  |  |  |  |  |  |  |  |  |  |  |  |  |  |
| Key worker | No | Base |  |  | **<0.001** |  | Base |  |  | **<0.001** | | Base |  |  | **<0.001** |
|  | Yes - Health care | 0.59 | 0.23 | 0.01 |  |  | 0.49 | 0.12 | 0.00 |  |  | 0.38 | 0.09 | 0.00 |  |
|  | Yes - Other key worker | 1.20 | 0.21 | 0.00 |  |  | 0.52 | 0.14 | 0.00 |  |  | 0.21 | 0.11 | 0.06 |  |
|  | Other | -14.39 | 902.09 | 0.99 |  |  | -14.74 | 547.15 | 0.98 |  |  | -1.92 | 1.00 | 0.06 |  |
|  |  |  |  |  |  |  |  |  |  |  |  |  |  |  |  |
| Employment | Full time | Base |  |  | **<0.001** |  | Base |  |  | **<0.001** | | Base |  |  | **<0.001** |
|  | Not seek work | -1.01 | 1.01 | 0.32 |  |  | -0.37 | 0.41 | 0.37 |  |  | -1.88 | 0.58 | 0.00 |  |
|  | Part work | 0.78 | 0.22 | 0.00 |  |  | 0.82 | 0.12 | 0.00 |  |  | 0.19 | 0.11 | 0.10 |  |
|  | Retired | -0.02 | 0.40 | 0.97 |  |  | 0.02 | 0.21 | 0.91 |  |  | 0.25 | 0.12 | 0.04 |  |
|  | Studying | -0.62 | 0.46 | 0.18 |  |  | -1.19 | 0.34 | 0.00 |  |  | -0.55 | 0.16 | 0.00 |  |
|  | Unemployed and seeking work | -0.50 | 0.72 | 0.49 |  |  | -0.42 | 0.36 | 0.24 |  |  | -0.23 | 0.22 | 0.30 |  |
|  |  |  |  |  |  |  |  |  |  |  |  |  |  |  |  |
| Work status | Work from home | Base |  |  | **<0.001** |  | Base |  |  | **<0.001** | | Base |  |  | **<0.001** |
|  | Work as usual | 1.25 | 0.24 | 0.00 |  |  | 2.05 | 0.16 | < 2e-16 |  |  | 1.42 | 0.10 | < 2e-16 |  |
|  | Laidoff | 0.82 | 0.33 | 0.01 |  |  | 0.44 | 0.28 | 0.12 |  |  | 0.61 | 0.15 | 0.00 |  |
|  | Other | 0.59 | 0.31 | 0.05 |  |  | 1.28 | 0.19 | 0.00 |  |  | 0.78 | 0.12 | 0.00 |  |
|  |  |  |  |  |  |  |  |  |  |  |  |  |  |  |  |
| Live with others | No | Base |  |  |  |  | Base |  |  |  |  | Base |  |  |  |
|  | Yes | 0.61 | 0.35 | **0.08** |  |  | 0.53 | 0.18 | **<0.001** |  |  | -0.01 | 0.10 | 0.91 |  |
|  |  |  |  |  |  |  |  |  |  |  |  |  |  |  |  |
| Live with 4y or younger | No | Base |  |  |  |  | Base |  |  |  |  | Base |  |  |  |
|  | Yes | -0.57 | 0.42 | **0.18** |  |  | -1.72 | 0.45 | **<0.001** |  |  | -1.62 | 0.29 | **<0.001** |  |
|  |  |  |  |  |  |  |  |  |  |  |  |  |  |  |  |
| Live with 5y - 18y | No | Base |  |  |  |  | Base |  |  |  |  | Base |  |  |  |
|  | Yes | -0.70 | 0.35 | **0.04** |  |  | -0.83 | 0.21 | **<0.001** |  |  | -0.20 | 0.11 | **0.07** |  |
|  |  |  |  |  |  |  |  |  |  |  |  |  |  |  |  |
| Live with 19 - 64 y | No | Base |  |  |  |  | Base |  |  |  |  | Base |  |  |  |
|  | Yes | 0.22 | 0.25 | 0.38 |  |  | 0.23 | 0.14 | **0.10** |  |  | 0.05 | 0.09 | 0.60 |  |
|  |  |  |  |  |  |  |  |  |  |  |  |  |  |  |  |
| Live with 65y or greater | No | Base |  |  |  |  | Base |  |  |  |  | Base |  |  |  |
|  | Yes | 0.08 | 0.35 | 0.83 |  |  | 0.11 | 0.19 | 0.58 |  |  | -0.56 | 0.17 | **<0.001** |  |
|  |  |  |  |  |  |  |  |  |  |  |  |  |  |  |  |
| Guardian of children under 18 y | No | Base |  |  |  |  | Base |  |  |  |  | Base |  |  |  |
|  | Yes | -0.54 | 0.28 | **0.06** |  |  | -0.93 | 0.20 | **<0.001** |  |  | -0.35 | 0.11 | **<0.001** |  |
|  |  |  |  |  |  |  |  |  |  |  |  |  |  |  |  |
| Think I have been already infected | No | Base |  |  | **0.00** |  | Base |  |  | **<0.001** | | Base |  |  | **<0.001** |
|  | Yes | -0.66 | 0.30 | 0.03 |  |  | 0.36 | 0.15 | 0.01 |  |  | 0.24 | 0.09 | **0.01** |  |
|  | Don't know | -0.71 | 0.27 | 0.01 |  |  | 0.77 | 0.12 | 0.00 |  |  | -0.21 | 0.10 | **0.03** |  |
|  |  |  |  |  |  |  |  |  |  |  |  |  |  |  |  |
| Received Covid-19 test | No | Base |  |  |  |  | Base |  |  |  |  | Base |  |  |  |
|  | Yes | -0.76 | 0.27 | **<0.001** |  |  | -0.42 | 0.14 | **<0.001** |  |  | 0.05 | 0.08 | 0.52 |  |
|  |  |  |  |  |  |  |  |  |  |  |  |  |  |  |  |
| I tested positive for Covid-19 | No | Base |  |  | **0.01** |  | Base |  |  | **0.01** |  | Base |  | **<0.001** |  |
|  | Yes | 1.16 | 0.63 | 0.07 |  |  | 0.39 | 0.43 | 0.36 |  |  | 1.25 | 0.19 | **<0.001** |  |
|  | Not applicable (not received test) | 0.79 | 0.28 | 0.004 |  |  | 0.41 | 0.14 | 0.00 |  |  | 0.09 | 0.09 | 0.32 |  |
|  |  |  |  |  |  |  |  |  |  |  |  |  |  |  |  |
| Housemate received Covid-19 test | No |  |  |  | 0.55 |  | Base |  |  | **0.01** |  | Base |  | 0.21 |  |
|  | Yes | -0.25 | 0.24 | 0.29 |  |  | 0.10 | 0.12 | 0.42 |  |  | 0.04 | 0.09 | 0.65 |  |
|  | Not applicable (living alone) | -0.11 | 0.28 | 0.70 |  |  | -0.50 | 0.19 | 0.01 |  |  | 0.18 | 0.10 | 0.07 |  |
|  |  |  |  |  |  |  |  |  |  |  |  |  |  |  |  |
| Housemate tested positive for Covid-19 | No | Base |  |  | **0.09** |  | Base |  |  | **<0.001** | | Base |  | **0.01** |  |
|  | Yes | -1.21 | 1.01 | 0.23 |  |  | -1.73 | 0.71 | 0.01 |  |  | -0.53 | 0.29 | 0.07 |  |
|  | Not applicable | -0.38 | 0.23 | 0.10 |  |  | -0.76 | 0.15 | **<0.001** |  |  | -0.20 | 0.09 | 0.02 |  |
|  |  |  |  |  |  |  |  |  |  |  |  |  |  |  |  |
| Currently have symptoms- cough | No | Base |  |  |  |  | Base |  |  |  |  | Base |  |  |  |
|  | Yes | -0.18 | 0.71 | 0.80 |  |  | -1.73 | 1.00 | **0.08** |  |  | -15.65 | 446.74 | 0.97 |  |
|  |  |  |  |  |  |  |  |  |  |  |  |  |  |  |  |
| Currently have symptoms- blocked nose | No | Base |  |  |  |  | Base |  |  |  |  | Base |  |  |  |
|  | Yes | -0.62 | 0.46 | **0.18** |  |  | -1.77 | 0.50 | **<0.001** |  |  | -0.59 | 0.20 | **0.003** |  |
|  |  |  |  |  |  |  |  |  |  |  |  |  |  |  |  |
| Currently have symptoms- feeling unusually tired | No | Base |  |  |  |  | Base |  |  |  |  | Base |  |  |  |
|  | Yes | -0.64 | 0.39 | **0.10** |  |  | -1.56 | 0.34 | **<0.001** |  |  | -0.69 | 0.16 | **<0.001** |  |
|  |  |  |  |  |  |  |  |  |  |  |  |  |  |  |  |
| Currently have symptoms- sore throat | No | Base |  |  |  |  | Base |  |  |  |  | Base |  |  |  |
|  | Yes | -0.11 | 0.39 | 0.77 |  |  | -0.15 | 0.22 | 0.50 |  |  | -0.44 | 0.17 | **0.01** |  |
|  |  |  |  |  |  |  |  |  |  |  |  |  |  |  |  |
| Currently have symptoms- shortness of breath | No | Base |  |  |  |  | Base |  |  |  |  | Base |  |  |  |
|  | Yes | -1.46 | 1.00 | **0.15** |  |  | -1.21 | 0.50 | **0.02** |  |  | -0.65 | 0.27 | **0.02** |  |
|  |  |  |  |  |  |  |  |  |  |  |  |  |  |  |  |
| Currently have symptoms- aches and pains | No | Base |  |  |  |  | Base |  |  |  |  | Base |  |  |  |
|  | Yes | -0.70 | 0.51 | **0.17** |  |  | -0.18 | 0.22 | 0.41 |  |  | -0.08 | 0.14 | 0.54 |  |
|  |  |  |  |  |  |  |  |  |  |  |  |  |  |  |  |
| Currently have symptoms- loss of sense of smell | No | Base |  |  |  |  | Base |  |  |  |  | Base |  |  |  |
|  | Yes | -13.79 | 773.78 | 0.99 |  |  | -13.93 | 469.32 | 0.98 |  |  | -1.33 | 1.00 | 0.18 |  |
|  |  |  |  |  |  |  |  |  |  |  |  |  |  |  |  |
| Currently have at least one of symptoms above | Yes | Base |  |  |  |  | Base |  |  |  |  | Base |  |  |  |
|  | No (no symptoms) | 0.81 | 0.26 | **0.002** |  |  | 0.71 | 0.14 | **<0.001** |  |  | 0.46 | 0.09 | **<0.001** |  |
|  |  |  |  |  |  |  |  |  |  |  |  |  |  |  |  |
| Trust test results for Covid-19 | | -0.29 | 0.09 | **0.002** |  |  | -0.16 | 0.06 | **0.01** |  |  | -0.17 | 0.04 | **<0.001** |  |
|  |  |  |  |  |  |  |  |  |  |  |  |  |  |  |  |
| Likely to be infected with Covid-19 in the next 4 weeks |  | 0.03 | 0.05 | 0.54 |  |  | 0.10 | 0.03 | **<0.001** |  |  | -0.08 | 0.02 | **<0.001** |  |

^1^ p value from log-likelihood ratio test

Table S6. Multivariable analysis and sensitivity analysis for avoiding crowds

|  |  |  | **Avoiding crowds** | | |  | **Avoiding crowds (cut-off change)** | | |  | **Avoiding crowds (Date)** | | |  | **Avoiding crowds (proportional odds)** | | |
| --- | --- | --- | --- | --- | --- | --- | --- | --- | --- | --- | --- | --- | --- | --- | --- | --- | --- |
| Variable | Level |  | Coefficient (log odds) | SE | P value |  | Coefficient (log odds) | SE | P value |  | Coefficient (log odds) | SE | P value |  | Coefficient (log odds) | SE | P value |
| Intercept |  |  | 0.24 | 0.71 | 0.7363 |  | 1.15 | 0.57 | 0.0435 |  | -0.10 | 0.76 | 0.8914 |  |  |  |  |
|  |  |  |  |  |  |  |  |  |  |  |  |  |  |  |  |  |  |
| Chance |  |  | -0.77 | 0.32 | **0.0154** |  | -0.27 | 0.24 | 0.2603 |  | -0.57 | 0.34 | 0.0971 |  | -0.45 | 0.22 | **0.04** |
|  |  |  |  |  |  |  |  |  |  |  |  |  |  |  |  |  |  |
| Age | 25-34 |  |  |  |  |  |  |  |  |  |  |  |  |  |  |  |  |
|  | 35-44 |  | 0.25 | 0.57 | 0.6627 |  | 0.47 | 0.40 | 0.248 |  | 0.24 | 0.60 | 0.6851 |  | 0.40 | 0.36 | 0.27 |
|  | 45-54 |  | 0.87 | 0.68 | 0.2008 |  | 0.42 | 0.52 | 0.4161 |  | 0.70 | 0.70 | 0.3166 |  | 0.59 | 0.48 | 0.22 |
|  | 55-64 |  | 1.09 | 0.54 | **0.0457** |  | 1.18 | 0.50 | 0.0189 |  | 0.62 | 0.61 | 0.3061 |  | 1.07 | 0.40 | **0.01** |
|  | 65+ |  | 1.38 | 0.81 | 0.0891 |  | 1.06 | 0.80 | 0.1812 |  | 1.52 | 0.85 | 0.0736 |  | 1.13 | 0.66 | 0.09 |
|  | Under 25 |  | 0.90 | 0.81 | 0.2623 |  | -0.47 | 0.55 | 0.3948 |  | 0.56 | 0.91 | 0.5406 |  | -0.29 | 0.54 | 0.59 |
| Gender |  |  |  |  |  |  |  |  |  |  |  |  |  |  |  |  |  |
|  | Male |  | -1.59 | 0.56 | **0.0048** |  | -0.54 | 0.33 | 0.1034 |  | -1.35 | 0.58 | **0.0196** |  | -0.80 | 0.30 | **0.01** |
|  | Other |  | NA |  |  |  | NA |  |  |  | NA |  |  |  | -0.11 | 1.66 | 0.95 |
|  |  |  |  |  |  |  |  |  |  |  |  |  |  |  |  |  |  |
| Work status | Work from home |  |  |  |  |  |  |  |  |  |  |  |  |  |  |  |  |
|  | Work as usual |  | -2.47 | 0.80 | **0.002** |  | -0.98 | 0.36 | **0.0066** |  | -2.34 | 0.81 | **0.0038** |  | -1.14 | 0.33 | **0.0006** |
|  | Laidoff |  | -0.06 | 0.63 | 0.9192 |  | -0.24 | 0.52 | 0.6435 |  | -0.21 | 0.66 | 0.7515 |  | -0.10 | 0.47 | 0.84 |
|  | Other |  | -0.12 | 0.51 | 0.8198 |  | -0.22 | 0.49 | 0.6573 |  | -0.26 | 0.58 | 0.6465 |  | 0.00 | 0.41 | 0.99 |
|  |  |  |  |  |  |  |  |  |  |  |  |  |  |  |  |  |  |
| Live with 5y - 18y | No |  |  |  |  |  |  |  |  |  |  |  |  |  |  |  |  |
|  | Yes |  | 1.16 | 0.59 | **0.05** |  | 0.54 | 0.50 | 0.2773 |  | 1.18 | 0.61 | 0.0529 |  | 0.81 | 0.44 | 0.07 |

Table S7. Multivariable analysis and sensitivity analysis for handwashing

|  |  |  | **Wash hands** | | |  | **Wash hands (Date)** | | |  | **Wash hands (proportional odds)** | | |
| --- | --- | --- | --- | --- | --- | --- | --- | --- | --- | --- | --- | --- | --- |
| Variable | Level |  | Coefficient (log odds) | SE | P value |  | Coefficient (log odds) | SE | P value |  | Coefficient (log odds) | SE | P value |
| Intercept |  |  | 2.22 | 1.37 | 0.104 |  | 1.92 | 1.51 | 0.2046 |  |  |  |  |
|  |  |  |  |  |  |  |  |  |  |  |  |  |  |
| Chance |  |  | -1.06 | 0.36 | **0.003** |  | -1.03 | 0.41 | **0.0128** |  | -1.07 | 0.33 | **0.001** |
|  |  |  |  |  |  |  |  |  |  |  |  |  |  |
| External |  |  | 0.62 | 0.29 | **0.029** |  | 0.73 | 0.31 | **0.0197** |  | 0.58 | 0.27 | **0.028** |
|  |  |  |  |  |  |  |  |  |  |  |  |  |  |
| Age | 25-34 |  |  |  |  |  |  |  |  |  |  |  |  |
|  | 35-44 |  | 0.74 | 0.59 | 0.210 |  | 1.55 | 0.74 | **0.0366** |  | 0.70 | 0.57 | 0.221 |
|  | 45-54 |  | -0.57 | 0.68 | 0.404 |  | -0.91 | 0.73 | 0.2136 |  | -0.79 | 0.65 | 0.219 |
|  | 55-64 |  | -0.36 | 0.69 | 0.601 |  | -0.74 | 0.83 | 0.3728 |  | -0.35 | 0.68 | 0.607 |
|  | 65+ |  | -0.78 | 1.46 | 0.591 |  | -1.01 | 1.62 | 0.532 |  | -0.66 | 1.44 | 0.645 |
|  | Under 25 |  | -1.40 | 0.83 | 0.090 |  | -1.50 | 0.92 | 0.1034 |  | -1.29 | 0.73 | 0.079 |
|  |  |  |  |  |  |  |  |  |  |  |  |  |  |
| Gender | Female |  |  |  |  |  |  |  |  |  |  |  |  |
|  | Male |  | -1.16 | 0.44 | **0.008** |  | -1.13 | 0.49 | **0.0208** |  | -1.11 | 0.41 | **0.006** |
|  | Other |  | NA |  |  |  | NA |  |  |  | NA |  |  |
|  |  |  |  |  |  |  |  |  |  |  |  |  |  |
| Key worker | No |  |  |  |  |  |  |  |  |  |  |  |  |
|  | Yes - Health care |  | -1.44 | 0.61 | **0.018** |  | -1.87 | 0.72 | **0.0091** |  | -1.40 | 0.56 | **0.013** |
|  | Yes - Other key worker |  | -0.73 | 0.62 | 0.238 |  | -1.02 | 0.72 | 0.153 |  | -0.32 | 0.59 | **0.591** |
|  | Other |  | NA |  |  |  | NA |  |  |  | NA |  |  |
|  |  |  |  |  |  |  |  |  |  |  |  |  |  |
| Work status | Work from home |  |  |  |  |  |  |  |  |  |  |  |  |
|  | Work as usual |  | 0.88 | 0.55 | 0.109 |  | 0.78 | 0.62 | 0.2082 |  | 0.75 | 0.52 | 0.146 |
|  | Laidoff |  | 0.19 | 0.77 | 0.807 |  | -0.05 | 0.84 | 0.9521 |  | 0.03 | 0.73 | 0.968 |
|  | Other |  | -2.80 | 0.91 | **0.002** |  | -3.77 | 1.24 | **0.0023** |  | -2.21 | 0.73 | **0.002** |
|  |  |  |  |  |  |  |  |  |  |  |  |  |  |
| Employment | Full time |  |  |  |  |  |  |  |  |  |  |  |  |
|  | Not seek work |  | 2.61 | 1.58 | 0.098 |  | 3.29 | 1.85E+00 | 0.0756 |  | 2.22 | 1.42E+00 | 0.118 |
|  | Part work |  | -0.30 | 0.57 | 0.600 |  | 1.07 | 0.77 | 0.1634 |  | -0.49 | 0.53 | 0.349 |
|  | Retired |  | 3.00 | 1.47 | **0.040** |  | 3.83 | 1.81 | **0.0344** |  | 2.20 | 1.34 | 0.099 |
|  | Studying |  | 2.30 | 1.21 | 0.058 |  | 3.43 | 1.66 | **0.0387** |  | 1.38 | 0.99 | 0.163 |
|  | Unemployed and seeking work | | NA |  |  |  | NA |  |  |  | NA |  |  |
|  |  |  |  |  |  |  |  |  |  |  |  |  |  |
| Live with 4y or younger | No |  |  |  |  |  |  |  |  |  |  |  |  |
|  | Yes |  | -1.55 | 0.71 | **0.029** |  | -1.21 | 0.85 | 0.1544 |  | -1.49 | 0.66 | **0.023** |
|  |  |  |  |  |  |  |  |  |  |  |  |  |  |
| Live with 5y - 18y | No |  |  |  |  |  |  |  |  |  |  |  |  |
|  | Yes |  | 1.72 | 0.79 | **0.030** |  | 1.64 | 0.96 | **0.0888** |  | 1.50 | 0.77 | **0.050** |
|  |  |  |  |  |  |  |  |  |  |  |  |  |  |
| Currently have symptoms- shortness of breath | No |  |  |  |  |  |  |  |  |  |  |  |  |
|  | Yes |  | -2.00 | 0.90 | **0.026** |  | -2.32 | 1.10 | **0.0359** |  | -2.00 | 0.86 | **0.020** |

Table S8. Multivariable analysis and sensitivity analysis for mask wearing

|  |  |  | **Wear masks** | | |  | **Wear masks (cut-off)** | | |  | **Wear masks (Date)** | | |  | **Wear masks (proportional odds)** | | |
| --- | --- | --- | --- | --- | --- | --- | --- | --- | --- | --- | --- | --- | --- | --- | --- | --- | --- |
| Variable | Level |  | Coefficient (log odds) | SE | P value | | Coefficient (log odds) | SE | P value |  | Coefficient (log odds) | SE | P value | | Coefficient (log odds) | SE | P value |
| Intercept |  |  | -2.06 | 1.34 | 0.1239 |  | 0.05 | 1.10 | 0.9645 |  | -0.79 | 1.60 | 0.623 |  |  |  |  |
|  |  |  |  |  |  |  |  |  |  |  |  |  |  |  |  |  |  |
| Chance |  |  | -0.54 | 0.27 | **0.0464** |  | -0.15 | 0.23 | 0.52 |  | -0.83 | 0.32 | **0.011** |  | -0.33 | 0.20 | 0.1059 |
|  |  |  |  |  |  |  |  |  |  |  |  |  |  |  |  |  |  |
| External |  |  | 0.53 | 0.28 | 0.0539 |  | 0.33 | 0.22 | 0.1322 |  | 0.40 | 0.32 | 0.216 |  | 0.38 | 0.20 | 0.0634 |
|  |  |  |  |  |  |  |  |  |  |  |  |  |  |  |  |  |  |
| PC1 |  |  | -0.46 | 0.20 | **0.0179** |  | -0.52 | 0.16 | **0.0014** |  | -0.47 | 0.22 | **0.035** |  | -0.46 | 0.14 | 0.0014 |
|  |  |  |  |  |  |  |  |  |  |  |  |  |  |  |  |  |  |
| Age | 25-34 |  |  |  |  |  |  |  |  |  |  |  |  |  |  |  |  |
|  | 35-44 |  | 0.24 | 0.46 | 0.6065 |  | -0.39 | 0.41 | 0.334 |  | 0.71 | 0.52 | 0.171 |  | -0.07 | 0.36 | 0.8509 |
|  | 45-54 |  | -0.37 | 0.62 | 0.5473 |  | -0.74 | 0.50 | 0.1369 |  | -0.64 | 0.73 | 0.378 |  | -0.44 | 0.45 | 0.3247 |
|  | 55-64 |  | -0.41 | 0.54 | 0.4503 |  | -1.23 | 0.48 | 0.0099 |  | -0.69 | 0.67 | 0.305 |  | -0.96 | 0.45 | 0.0331 |
|  | 65+ |  | -0.50 | 0.85 | 0.557 |  | -0.71 | 0.65 | 0.2796 |  | -1.23 | 1.13 | 0.278 |  | -0.66 | 0.61 | 0.2723 |
|  | Under 25 |  | -0.31 | 0.64 | 0.6247 |  | 0.43 | 0.65 | 0.5043 |  | -0.67 | 0.72 | 0.355 |  | 0.09 | 0.49 | 0.8572 |
|  |  |  |  |  |  |  |  |  |  |  |  |  |  |  |  |  |  |
| Gender | Female |  |  |  |  |  |  |  |  |  |  |  |  |  |  |  |  |
|  | Male |  | -0.04 | 0.39 | 0.9195 |  | 0.17 | 0.34 | 0.6182 |  | 0.32 | 0.43 | 0.467 |  | 0.12 | 0.29 | 0.687 |
|  | Other |  | NA |  |  |  | NA |  |  |  | NA |  |  |  | NA |  |  |
|  |  |  |  |  |  |  |  |  |  |  |  |  |  |  |  |  |  |
| Key worker | No |  |  |  |  |  |  |  |  |  |  |  |  |  |  |  |  |
|  | Yes - Health care |  | -1.01 | 0.55 | 0.067 |  | -0.09 | 0.39 | 0.8144 |  | -0.92 | 0.58 | 0.111 |  | -0.27 | 0.34 | 0.432 |
|  | Yes - Other key worker |  | 0.66 | 0.47 | 0.1593 |  | -0.20 | 0.44 | 0.6426 |  | 0.36 | 0.54 | 0.513 |  | 0.16 | 0.40 | 0.6914 |
|  | Other |  | -0.25 | 1.29 | 0.8438 |  | -1.71 | 1.21 | 0.1583 |  | 0.23 | 1.37 | 0.864 |  | -1.31 | 1.21 | 0.2789 |
|  |  |  |  |  |  |  |  |  |  |  |  |  |  |  |  |  |  |
| Income | <24400 |  |  |  |  |  |  |  |  |  |  |  |  |  |  |  |  |
|  | <33600 |  | 0.25 | 0.53 | 0.6432 |  | -0.37 | 0.46 | 0.4237 |  | 0.01 | 0.59 | 0.989 |  | -0.17 | 0.40 | 0.6631 |
|  | <41100 |  | -0.11 | 0.57 | 0.8444 |  | -0.67 | 0.48 | 0.1562 |  | -0.96 | 0.68 | 0.157 |  | -0.47 | 0.42 | 0.2582 |
|  | Above 41100 |  | -0.15 | 0.55 | 0.7874 |  | -0.12 | 0.47 | 0.7919 |  | -0.73 | 0.62 | 0.244 |  | -0.25 | 0.40 | 0.5284 |
|  | Prefer not to answer |  | 1.37 | 0.58 | **0.0173** |  | 0.27 | 0.55 | 0.627 |  | 1.10 | 0.63 | 0.084 |  | 0.72 | 0.49 | 0.1413 |

Table S9. Multivariable analysis for contacts

|  |  | **Physical contact** | | |  | **Non-physical within 1m** | | |  | **Non-physical >1m** | | |
| --- | --- | --- | --- | --- | --- | --- | --- | --- | --- | --- | --- | --- |
| Variable | Level | Coefficient (log count) | SE | P value |  | Coefficient (log count) | SE | P value |  | Coefficient (log count) | SE | P value |
| Intercept |  | -0.85 | 0.56 | 0.128 |  | -1.55 | 0.54 | 0.004 |  | -0.49 | 0.41 | 0.238 |
|  |  |  |  |  |  |  |  |  |  |  |  |  |
| Chance |  | -0.11 | 0.23 | 0.624 |  | 0.35 | 0.21 | 0.096 |  | 0.36 | 0.17 | **0.031** |
|  |  |  |  |  |  |  |  |  |  |  |  |  |
| Age | 25-34 | Base |  |  |  | Base |  |  |  | Base |  |  |
|  | 35-44 | -1.01 | 0.47 | **0.032** |  | 0.04 | 0.37 | 0.913 |  | 0.18 | 0.27 | 0.497 |
|  | 45-54 | 0.00 | 0.50 | 0.994 |  | 0.01 | 0.46 | 0.982 |  | 0.63 | 0.36 | 0.084 |
|  | 55-64 | 0.58 | 0.44 | 0.189 |  | -0.14 | 0.42 | 0.731 |  | 0.33 | 0.32 | 0.303 |
|  | 65+ | 0.78 | 0.66 | 0.240 |  | -0.18 | 5.92E-01 | 0.761 |  | -0.40 | 0.47 | 0.395 |
|  | Under 25 | 0.89 | 0.53 | 0.094 |  | 0.07 | 0.45 | 0.869 |  | 0.17 | 0.35 | 0.639 |
|  |  |  |  |  |  |  |  |  |  |  |  |  |
| Gender | Female | Base |  |  |  | Base |  |  |  | Base |  |  |
|  | Male | -0.26 | 0.35 | 0.459 |  | 0.28 | 0.28 | 0.309 |  | 0.34 | 0.22 | 0.127 |
|  | Other | -0.66 | 1.64E+00 | 0.690 |  | 0.51 | 1.46E+00 | 0.725 |  | NA |  |  |
|  |  |  |  |  |  |  |  |  |  |  |  |  |
| Key worker | No | Base |  |  |  |  |  |  |  |  |  |  |
|  | Yes - Health care | 0.96 | 0.40 | **0.015** |  |  |  |  |  |  |  |  |
|  | Yes - Other key worker | 1.19 | 0.42 | **0.004** |  |  |  |  |  |  |  |  |
|  | Other | -36.02 | 3.36E+07 | 1.000 |  |  |  |  |  |  |  |  |
|  |  |  |  |  |  |  |  |  |  |  |  |  |
| Work status | Work from home |  |  |  |  | Base |  |  |  | Base |  |  |
|  | Work as usual |  |  |  |  | 2.03 | 0.32 | **< 0.001** |  | 1.52 | 0.24 | **< 0.001** |
|  | Laidoff |  |  |  |  | 0.57 | 0.47 | 0.232 |  | -0.45 | 0.39 | 0.243 |
|  | Other |  |  |  |  | 1.39 | 0.41 | **0.001** |  | 0.94 | 0.31 | **0.002** |
|  |  |  |  |  |  |  |  |  |  |  |  |  |
| Ethnicity | White - British |  |  |  |  |  |  |  |  | Base |  |  |
|  | White - Other |  |  |  |  | -0.88 | 1.64 | 0.590 |  | 0.53 | 1.16 | 0.651 |
|  | Other |  |  |  |  | 5.52 | 1.94 | **0.004** |  | 4.77 | 1.63 | **0.004** |
|  |  |  |  |  |  |  |  |  |  |  |  |  |
| Guardian | No |  |  |  |  |  |  |  |  |  |  |  |
|  | Yes |  |  |  |  | -1.05 | 0.42 | **0.012** |  |  |  |  |
|  |  |  |  |  |  |  |  |  |  |  |  |  |
| Live with 5y - 18y | No |  |  |  |  |  |  |  |  | Base |  |  |
|  | Yes |  |  |  |  |  |  |  |  | -1.25 | 0.33 | **< 0.001** |
|  |  |  |  |  |  |  |  |  |  |  |  |  |
| Interaction | Chance:Ethnic White - other | |  |  |  | 0.66 | 0.85 | 0.435 |  | 0.14 | 0.61 | 0.818 |
|  | Chance:Ethnic Other |  |  |  |  | -2.25 | 0.91 | **0.013** |  | -2.41 | 0.77 | **0.002** |

**4.4 Contacts**

The number of physical contacts was not associated with Chance score in the univariable or multivariable analysis (Count ratio (CR) 0.90, 95%CI 0.58 – 1.39, p = 0.62). For both non-physical contacts with and without distancing, higher Chance score was associated with a larger number of contacts made in both univariable and multivariable models. The final multivariable models for these two contact types included an interaction term between Chance score and ethnicity (Table S10). For white British, a unit increase in Chance score increased the number of non-physical contacts without distancing and distancing 1.42 (95%CI 0.94 – 2.13, p = 0.1) and 1.43 (95%CI 1.03 – 1.98, p = 0.03) times, respectively. For non-white groups, an opposite association was identified, where a unit increase in Chance score substantially reduced the number of these contacts. No associations were identified for white non-British. Powerful others score was negatively associated with all contact types univariably, however, none of these effects remained significant in the multivariable analysis. A univariable association between Internal score and non-physical contacts without distancing also disappeared in the multivariable analysis. PC2 was positively associated with the number of non-physical contacts with distancing univariably but did not remain in the final model.

Table S10. The effect of Chance variable across different ethnic groups on the three types of contact derived from the final multivariable negative binomial regression models

|  | **Physical contact**^2^ | | | |  | **Non-physical within 1m**^3^ | | | |  | **Non-physical >1m**^4^ | | | |
| --- | --- | --- | --- | --- | --- | --- | --- | --- | --- | --- | --- | --- | --- | --- |
|  | aCR^1^ | 2.5% | 97.5% | p value |  | aCR | 2.5% | 97.5% | p value |  | aCR | 2.5% | 97.5% | p value |
| White British | 0.90 | 0.58 | 1.39 | 0.62 |  | 1.42 | 0.94 | 2.13 | 0.10 |  | 1.43 | 1.03 | 1.98 | 0.03 |
| White Other |  |  |  |  |  | 2.75 | 0.54 | 13.91 | 0.22 |  | 1.64 | 0.51 | 5.26 | 0.40 |
| Other |  |  |  |  |  | 0.15 | 0.03 | 0.85 | 0.03 |  | 0.13 | 0.03 | 0.57 | 0.01 |

^1^ Adjusted count ratio
^2^ The final model included Age, Gender, Key worker.
^3^ The final model included Age, Gender, Work status, Ethnicity, Guardian status and interaction between Chance and Ethnicity.
^4^ The final model included Age, Gender, Work status, Ethnicity, live with 5 – 18 years old individual and interaction between Chance and Ethnicity

**4.5 Prediction of Chance score**

In the univariable analysis for Chance score, work status (p < 0.001), gender (p = 0.02), ethnicity (p = 0.1), and education (p = 0.04) were significant at an alpha level 0.1. None of clinical symptoms were associated with Chance score at this significance level. In the final multivariable model, income, work status and ethnicity remained in addition to two a priori confounders age and gender. Associations between these five variables were visualised using multiple correspondence analysis (Figure 3A). Non-British white ethnic group and working from home located closely, suggesting an association between these groups. Lower income and white British also located together. As shown in Figure 3B, Chance score was higher among those with lower income and working as usual or being laid-off. Non-British white had significantly lower Chance scores than white British and other ethnic groups (Table S11).

Table S11. Multivariable analysis for Chance score

|  |  | Coefficient | SE | p value |
| --- | --- | --- | --- | --- |
| Variable | Intercept | 2.07 | 0.08 | <2e-16 |
|  |  |  |  |  |
| Income | <24400 | Base |  |  |
|  | <33600 | -0.10 | 0.14 | 0.48 |
|  | <41100 | -0.33 | 0.14 | 0.02 |
|  | Above 41100 | -0.40 | 0.13 | 0.00 |
|  | Prefer not to answer | -0.23 | 0.15 | 0.14 |
|  |  |  |  |  |
| Ethnicity | White - British | Base |  |  |
|  | White - Other | -0.29 | 0.14 | 0.04 |
|  | Other | 0.02 | 0.16 | 0.91 |
|  |  |  |  |  |
| Gender | Female |  |  |  |
|  | Male | -0.02 | 0.10 | 0.84 |
|  | Other | -1.15 | 0.46 | 0.01 |
|  |  |  |  |  |
| Age | 25-34 | Base |  |  |
|  | 35-44 | 0.22 | 0.12 | 0.06 |
|  | 45-54 | 0.20 | 0.15 | 0.18 |
|  | 55-64 | 0.13 | 0.14 | 0.34 |
|  | 65+ | 0.33 | 0.21 | 0.12 |
|  | Under 25 | -0.08 | 0.17 | 0.62 |
|  |  |  |  |  |
| Work status | Work from home | Base |  |  |
|  | Work as usual | 0.23 | 0.11 | 0.03 |
|  | Laidoff | 0.25 | 0.16 | 0.11 |
|  | Other | -0.32 | 0.14 | 0.03 |


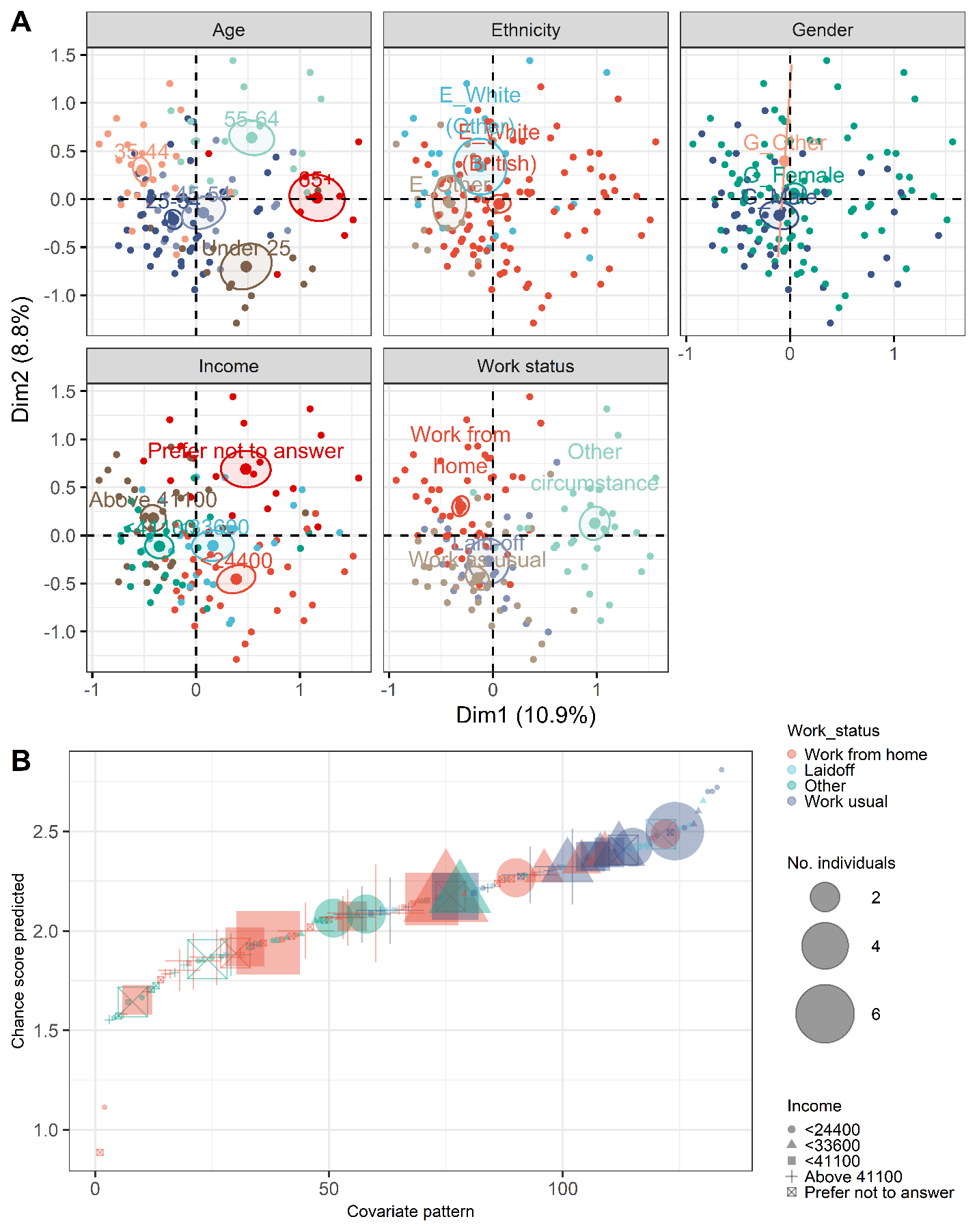
**Figure S5. Predicting Chance score across demographic and work status variables**(A) Multiple correspondence analysis visualising the association between each of five variables remained in the final model for Chance score. (B) Predicted Chance scores for each variable patterns stratified by work status (shown in different colours) and income (shown in different shapes).

**5. Discussion**

*5.1 Chance score*

Higher Chance score was associated with lower income groups and white British and non-white individuals. Previous studies similarly reported that individuals with a lower socio-economic status had higher luck perception ^3^ and fatalism ^22^. It is also reported that specific ethnic groups such as African Americans and Latinos have higher fatalism ^22,23^. Our results additionally suggested those who were working “as usual” (i.e. at the same workplace location) as before the pandemic had higher Chance scores compared to those working from home. These individuals had a greater number of contacts (after adjusting for other variables) probably because of their work circumstances, hence they might have felt inevitable to be exposed to the virus. Their work circumstances may also not allow them to take sick leave, which was reported to render individuals to associate SARS-CoV-2 with death ^24^. A study reported one’s fatalism towards SARS-CoV-2 may be altered through optimistic messaging ^25^, with implications for public communications during pandemics. Nevertheless, given that luck perception and fatalism are social constructs and stemmed out from structures ^26^, we argue that it is imperative to identify components of health systems that may facilitate fatalism in different contexts and address these in the post-Covid19 era.

*5.2 Role of Internal and Powerful others HLC variables on health behaviours*

There was little evidence to suggest an association between Internal locus of control and health protective behaviours or contact frequencies. Lack of direct association between Internal variable and sanitary measures for SARS-CoV-2 was also reported elsewhere ^27^. These findings contrast with other studies that reported higher Internal score was associated with healthier behaviours, particularly if they have strong health connotations ^28,29^. These disparities may indicate that the health behaviours in the context of a pandemic may be less influenced by an individual’s beliefs regarding the control they have over their own health outcomes. Alternatively, individuals with higher Internal score might have implemented other practices they believed effective. Regardless, these individuals tended to believe they were less susceptible to the SARS-CoV-2 infection (Supplementary Figure2).

Higher Powerful others locus of control was associated with more frequent handwashing and mask wearing (but not with contact patterns), possibly reflecting higher compliance with advice from the government or others such as doctors or family members. This study was conducted in August 2020 when control measures were easing (e.g. reopening of leisure centres) and before the ‘Rule of Six’, which restricted the size of social gatherings, was introduced in the UK. It may be that limiting contacts including avoiding crowds was perceived as less important and feasible by these individuals than handwashing and mask wearing.

*5.3 Dimension of trust and its role in protective behaviours*

The median trust score of the participants was found to be 2.4 out of 5. A previous study found that confidence in the government in England declined over the course of the pandemic, particularly since May 2020 ^30,31^. Principal component analysis (PCA) identified two dimensions of trust; general trust towards the government (PC1) and the perceived government’s interest in the public (PC2). PC1 but not PC2 was found to be negatively associated with the odds of always wearing a mask. This negative association was reported elsewhere, which measured trust towards politicians by single question ^32^. Inclusion of PC1 in the final model for mask wearing changed the coefficient of Chance score by 10% (from -0.6 to -0.54 after inclusion), suggesting that general trust is a moderate confounder for this outcome. We are not aware of other SARS-CoV-2 -related studies that explored the dimension of trust and its differential effect on health behaviours, which requires a lengthy list of questions. Given many of these questions loaded onto PC1, it is useful to collect information regarding participants’ trust towards government, even using a few questions, to adjust for this variable when examining variation in behaviours and compliance with guidelines.

*5.4 Study design*

We did not ask participants to report their behavioural changes during the pandemic, partly to keep the questionnaire of manageable length, but also because self-reported changes, particularly in a cross-sectional survey, are often unreliable and subject to recall bias. Indeed, the cross-sectional nature of our study design means that limited inference can be made regarding causality between luck perception and contact patterns and their changes, a crucial aspect for mechanistic models for human behavioural changes. Furthermore, luck perception itself may have altered in response to various factors such as epidemic status and policies during the pandemic. The advent of digital apps would greatly facilitate the collection of these information over time.

**6. Conclusions**

We provide evidence that luck perception may be a determinant of implementation of health protective behaviours and social contact patterns during infectious disease epidemics. Despite widespread availability and use of data on human mobility and contact patterns during the SARS-CoV-2 pandemic, our mechanistic understanding of individual and social factors influencing human contact and behavioural change remains limited. Studies on these factors during peacetime are indispensable for effective response to future pandemics.

**7. Reference**

1. Rotter J. Social learning and clinical psychology. - PsycNET. 1954 [cited 2021 Aug 20]; Available from: https://psycnet.apa.org/doiLanding?doi=10.1037%2F10788-000

2. Wallston KA, Strudler Wallston B, DeVellis R. Development of the Multidimensional Health Locus of Control (MHLC) Scales. Health Education Monographs. 1978 Mar 1;6(1):160–70.

3. Poortinga W, Dunstan FD, Fone DL. Health locus of control beliefs and socio-economic differences in self-rated health. Preventive Medicine. 2008 Apr 1;46(4):374–80.

4. Luszczynska A, Schwarzer R. Multidimensional Health Locus of Control: Comments on the Construct and its Measurement. J Health Psychol. 2005 Sep 1;10(5):633–42.

5. Hartke RJ, Kunce JT. Multidimensionality of health-related locus-of-control-scale items. Journal of Consulting and Clinical Psychology. 1982;50(4):594–5.

6. Sørlie T, Sexton HC. Predictors of change in health locus of control following surgical treatment. Personality and Individual Differences. 2004 Apr 1;36(5):991–1004.

7. Holman D, Lynch R, Reeves A. How do health behaviour interventions take account of social context? A literature trend and co-citation analysis. Health (London). 2018 Jul 1;22(4):389–410.

8. Baier A. Trust and Antitrust. Ethics. 1986 Jan 1;96(2):231–60.

9. Larson HJ, Clarke RM, Jarrett C, Eckersberger E, Levine Z, Schulz WS, et al. Measuring trust in vaccination: A systematic review. Human vaccines & Immunotherapeutics. 2018;14(7):1599–609.

10. Enticott G, Maye D, Fisher R, Ilbery B, Kirwan J. Badger Vaccination: Dimensions of Trust and Confidence in the Governance of Animal Disease. Environ Plan A. 2014 Dec 1;46(12):2881–97.

11. Poortinga W, Pidgeon NF. Exploring the Dimensionality of Trust in Risk Regulation. Risk Analysis. 2003;23(5):961–72.

12. Chan RK. Tackling COVID-19 risk in Hong Kong: Examining distrust, compliance and risk management. Current Sociology. 2021 Jul 1;69(4):547–65.

13. Metlay D. Institutional Trust and Confidence: A Journey into a Conceptual Quagmire. In: Social Trust and the Management of Risk. Routledge; 1999.

14. Earle TC, Cvetkovich G. Social trust and culture in risk management. In: Social Trust and the Management of Risk. Routledge; 1999.

15. Eames KTD, Tilston NL, Brooks-Pollock E, Edmunds WJ. Measured Dynamic Social Contact Patterns Explain the Spread of H1N1v Influenza. PLOS Computational Biology. 2012 Aug 3;8(3):e1002425.

16. Mossong J, Hens N, Jit M, Beutels P, Auranen K, Mikolajczyk R, et al. Social Contacts and Mixing Patterns Relevant to the Spread of Infectious Diseases. PLOS Medicine. 2008 Mar 25;5(3):e74.

17. Prem K, Cook AR, Jit M. Projecting social contact matrices in 152 countries using contact surveys and demographic data. PLoS Comput Biol [Internet]. 2017 Sep 12 [cited 2020 Apr 1];13(9). Available from: https://www.ncbi.nlm.nih.gov/pmc/articles/PMC5609774/

18. Gimma A, Munday JD, Wong KL, Coletti P, Zandvoort K van, Prem K, et al. CoMix: Changes in social contacts as measured by the contact survey during the COVID-19 pandemic in England between March 2020 and March 2021 [Internet]. 2021 May [cited 2021 Oct 26] p. 2021.05.28.21257973. Available from: https://www.medrxiv.org/content/10.1101/2021.05.28.21257973v1

19. Jarvis CI, Gimma A, van Zandvoort K, Wong KLM, Abbas K, Villabona-Arenas CJ, et al. The impact of local and national restrictions in response to COVID-19 on social contacts in England: a longitudinal natural experiment. BMC Medicine. 2021 Feb 19;19(1):52.

20. Anonymous. World Population Prospects - Population Division - United Nations [Internet]. 2019 [cited 2021 Oct 27]. Available from: https://population.un.org/wpp/Download/Standard/Interpolated/

21. Phoebe Dunn, Lucinda Allen, Genevieve Cameron, Akanksha Mimi Malhotra, Hugh Alderwick. COVID-19 policy tracker 2020 [Internet]. 2020 [cited 2021 Oct 1]. Available from: https://www.health.org.uk/news-and-comment/charts-and-infographics/covid-19-policy-tracker

22. Shen L, Condit CM, Wright L. The Psychometric Property and Validation of a Fatalism Scale. Psychol Health. 2009 Jun;24(5):597–613.

23. Chavez LR, Hubbell FA, Mishra SI, Valdez RB. The influence of fatalism on self-reported use of Papanicolaou smears. Am J Prev Med. 1997 Dec;13(6):418–24.

24. Jimenez T, Restar A, Helm PJ, Cross RI, Barath D, Arndt J. Fatalism in the context of COVID-19: Perceiving coronavirus as a death sentence predicts reluctance to perform recommended preventive behaviors. SSM - Population Health. 2020 Aug 1;11:100615.

25. Hayes J, Clerk L. Fatalism in the Early Days of the COVID-19 Pandemic: Implications for Mitigation and Mental Health. Frontiers in Psychology. 2021;12:2331.

26. Perfetti AR. Fate and the clinic: a multidisciplinary consideration of fatalism in health behaviour. Medical Humanities. 2018 Mar 1;44(1):59–62.

27. Nordfjaern T, Mehdizadeh M, Fallah Zavareh M. Social Psychology of Coronavirus Disease 2019: Do Fatalism and Comparative Optimism Affect Attitudes and Adherence to Sanitary Protocols? Frontiers in Psychology. 2021;12:1389.

28. Steptoe A, Wardle J. Locus of control and health behaviour revisited: A multivariate analysis of young adults from 18 countries. British Journal of Psychology. 2001;92(4):659–72.

29. Walsh A, Simpson EEA. Health cognitions mediate physical (in)activity and walking in midlife women. Maturitas. 2020 Jan 1;131:14–20.

30. Fancourt D, Steptoe A, Wright L. The Cummings effect: politics, trust, and behaviours during the COVID-19 pandemic. The Lancet. 2020 Aug 15;396(10249):464–5.

31. Reuters Institute for the Study of Journalism. Trust in UK government and news media COVID-19 information down, concerns over misinformation from government and politicians up [Internet]. Reuters Institute for the Study of Journalism. 2020 [cited 2021 Nov 2]. Available from: https://reutersinstitute.politics.ox.ac.uk/trust-uk-government-and-news-media-covid-19-information-down-concerns-over-misinformation

32. Jones P, Menon A, Hicken A, Rozek LS. Global adoption of personal and social mitigation behaviors during COVID-19: The role of trust & confidence. PLOS ONE. 2021 Aug 9;16(9):e0256159.

**8. Survey**

The original survey used can be found here: https://github.com/arata-hidano/Covid_survey_2020/blob/master/Trust_HLOC_survey_covid_final.pdf
